# The proportional Caputo operator approach to the thermal transport of Jeffery tri-hybrid nanofluid in a rotating frame with thermal radiation

**DOI:** 10.1038/s41598-023-29222-7

**Published:** 2023-08-23

**Authors:** Muhammad Arif, Poom Kumam, Wiboonsak Watthayu, Luca Di Persio

**Affiliations:** 1 Fixed Point Research Laboratory, Fixed Point Theory and Applications Research Group, Center of Excellence in Theoretical and Computational Science (TaCS-CoE), Faculty of Science, Thonburi (KMUTT), King Mongkut’s University of Technology, 126 Pracha Uthit Rd., Bang Mod, Thung Khru, Bangkok, 10140 Thailand; 2 Center of Excellence in Theoretical and Computational Science (TaCS-CoE), Faculty of Science, Thonburi (KMUTT), King Mongkut’s University of Technology, 126 Pracha Uthit Rd., Bang Mod, Thung Khru, Bangkok, 10140 Thailand; 3 Department of Computer Science, College of Mathematics, University of Verona, Verona, Italy

**Keywords:** Energy science and technology, Engineering, Materials science, Mathematics and computing, Nanoscience and technology

## Abstract

Engine Oil is a widely used fluid in engineering problems, particularly to enhance the rate of heat transfer when these working fluids play a fundamental role. We consider engine oil as a base fluid and the suspension of different shaped (Spherical cylindrical and platelet) nanoparticles dispersed uniformly in the base fluid to enhance the working capability of engine oil. The spherical shape ${\text{CuO}}$, platelet shape ${\text{Al}}_{2} {\text{O}}_{3}$ and cylindrical shape ${\text{TiO}}_{2}$ nanoparticles are added in engine oil to constitute tri-hybrid nanofluid aiming at obtaining better thermal performance. Furthermore, we also analyze the Jeffery tri-hybrid nanofluid in a rotating frame over an infinite vertical plate. More precisely, the classical model of Jeffery tri-hybrid nanofluid is transformed into a time-fractional model by applying the newly developed constant proportional Caputo fractional derivatives. Sharp numerical results are obtained applying a Laplace transform steered approach. All the flow parameters are highlighted through graphs via MATHCAD. Furthermore, a comparative analysis between nanofluid, hybrid nanofluid and tri-hybrid nanofluid has been performed showing that tri-hybrid nanofluid has good thermal performance. The solutions of the constant proportional operator are discussed classically by taking fractional parameter α → 1. Moreover, some engineering quantities have been calculated and presented in tables. During the analysis we dispersing the mixture of nanoparticles in engine oil base fluid enhanced the heat transfer up-to18.72% which can efficiently improve the lubricity of the engine oil.

## Introduction

In Nature, it is possible to recognize many different fluids. Their categorization allows classifying them into two main classes: Newtonian and non-Newtonian fluids. Non-Newtonian fluids such, e.g., blood, engine oil, grease, transformer oil, grease, silicone oil, vegetable oil as cutting fluid, paints, toothpaste, ketchup, drilling muds, etc., have attracted researchers' attention because of their applicability within a large set of heterogeneous applications in both engineering and modern technology. Because of their complex nature, many models have been specifically developed for each different scientific purpose. In the present analysis, we consider the Jeffery fluid model, since it represents one of the main non-Newtonian fluid models. It is worth to mention that the Jeffrey fluid is a special kinds of non-Newtonian fluid since it is characterized by having time derivative, instead of convective type derivative^1^. Moreover, this kind of fluid is known as viscoelastic with associated governing equations where the generalize two parameters as $\lambda_{1}$ and $\lambda_{2}$ represent, respectively, the parameter of retardation and relaxation time. Let us underline that Jeffry fluids have many real-life applications, spanning from Engineering, to Biology, but they are also characterized by low thermal transport properties. In particular, the first experimental work conducted by Choi^2^ in 1995, showed that by the suspension of nanometer sized solid particles in conventional fluid, base fluid thermal performance increase. Such result triggered many studies focused on considering different nanoparticles in the base fluid with the aim to improve thermal transport properties. There are many studies conducted for the evaluation of nanofluid and its applications in many fields of sciences. Like, Ali et al*.*^3^ calculated the effect of heat and mass transfer taking the nanoparticles in the base fluid. Furthermore, they have considered the impact of radiation, MHD and chemical reaction in their study. Pordanjani et al*.*^4^ discussed the thermal characteristics of nanofluid in heat exchangers for the purpose of saving energy. Jamei et al*.*^5^ highlighted the influence of nanofluids and specific heat capacity of host fluid for thermal applications in solar energy system. Bairwa et al*.*^6^ analyzed some modern applications of nanofluid and its impact on different engineering problems. Afterwards some of the thermal characteristics of nano-liquids were highlighted by Sheikholeslami^7^. Amoo et al*.*^8^ carried a review in which the authors discussed the nanoliquids unique applications especially in the transport phenomena. Tlili et al*.*^9^ inspected the applications of aluminum oxide nanoparticles in squeezing cooling processes. Arif et al*.*^10^ developed the enhancement in the heat transfer rate by considering the nanocomposites in the base fluid engine oil. Moreover, in this study they have considered the impact of non-uniform temperature at the plate.

In the heat transport phenomena nanofluid shown good results compared to regular fluid. Nevertheless, we need advance cooling properties which cannot be obtained from the simple nanofluid. For advance cooling purpose the mixing up of two particles of nano size dispersed in the host fluid shows promising enhancement in the heat transport phenomena. There are many practical uses of the hybrid nanofluid in engineering and medical sciences, like heat exchangers, heat pumps, heat turbines, cooling of electrical circuits, etc. Recently, Suresh et al*.*^11^ discussed the impact of aluminum and copper hybrid nano-liquids in water. In this study they developed laminar flow and the fluid circulate in a tube using hybrid nano-liquids for analyzing heat transport properties of the considered fluid. Similarly, Arif et al*.*^12^ carried a research where the authors highlight the thermal applications of maxwell fluid by considering the GO-MoS_2_ in engine oil and the fluid is considered to flow over an oscillating cylinder. During this study the authors explained theoretically, that the hybrid nanofluid performed well compared to regular engine oil and engine oil containing single nanoparticles. Furthermore, the research of hybrid nanofluid start using in many fields of sciences and performed well. Afterwards, Huminic^13^, Mousavi et al*.*^14^ and Dinarvand and Nejad^15^ investigated hybrid nanofluid in different thermal systems and heat exchangers. Form their studies they found that the hybrid nano-liquids is more efficient fluid in nanotechnologies compared to classical and nanofluids. Moreover, Lund et al*.*^16^ investigated thermal stable characteristics of solid hybrid nano-composite for the flow of mixed convection with slip effect. Alhadri et al*.*^17^ discussed the the impact of hybrid nano-composites using the artificial network techniques indifferent circumstances. Similarly, Yashkun et al*.*^18^ studied where the authors attracted by the unique thermal properties of hybrid nano-composites and they considered stretching sheet for the flow with joule heating effect. Some advance applications of hybrid nanofluid is given in the references^19–21^.

The study of dispersion of various kinds of nano size particles in the working host fluid which is referred as “tri-hybrid nanofluid” have enormous applications in modern science and nanotechnologies. It is very important to discuss here that not only particles effect heat transfer rate, but shape of particles is also play a vital role in heat transport performance. In this analysis the combination of three different nanoparticles with dissimilar shaped have been considered for the advance applications in medical sciences and engineering problems. Many researchers find out theoretically and experimentally that tri-hybrid nanofluid performance in thermal transport phenomena have good results compared to hybrid and mono nanofluid. The study related to tri-hybrid nanofluid was recently inspected by Arif et al*.*^22^. During this analysis they considered three various shaped of nanoparticles and the combination of different kinds of nanoparticles in the base fluid water for thermal performance of radiator. Animasaun et al*.*^23^ studied where the authors highlight the dynamics of tri-hybrid nanofluid with the combine impact of heat and magnetic effect. Sahoo^24^ discussed the dissimilar nanoparticles for the heat transfer and second law analysis using the various shape of nanoparticles. During the analysis Sahoo proved that the performance of tri-hybrid nanofluid perform well in the dynamics of fluid flow. Elnaqeeb et al*.*^25^ discussed the impact of tri-hybrid nano-liquids for various advance applications in dynamical system and industrial technologies. The fluid has three different kinds of nanoparticles with dissimilar shaped and densities for the advance cooling purposes. Adun et al*.*^26^ investigated tri-hybrid nano-liquid, its synthesis processes, analysis of stability of the fluid, thermal performance and heat transport phenomena of tri-hybrid nanofluid and also discussed its environmental effects on different systems. Recentyl, Ramadhan et al*.*^27^ investigated and highlighting thermos-physical properties of tri-hybrid nanofluid using experimental approach by taking water as base fluid and explain various physical applications of tri-hybrid nanofluid. Similarly, Manjunatha et al*.*^28^ discussed theoretically synthesis of convective heat transfer in try nano-liquids and considered the fluid passing through a stretching sheet. In another paper, Ramadhan et al*.*^29^ discussed the stability analysis of ternary nano-liquids in the base fluid water.

There are many problems of fluid flow in different domain. But the fluid in a rotating frame have been used in many physical situations due to its tremendous applications in engineering problems. Researchers are taking interest in the fluid is assumed to pass in a rotating frame due to its enormous applications like, Ramzan et al*.*^30^ considered and discussed hybrid nano-liquids with two kinds of nanoparticles CNT and MWCNT are considered in the fluid flow due to its wide range of industrial and engineering problems. Waqas et al*.*^31^ discussed the thermal analysis of hybrid nanofluid flow in a rotating channel using blood as a base fluid. Shoaib et al*.*^32^ investigated the influence of 3D flow with MHD effect considering hybrid nano-liquids and flow passes through rotating disk with the effect of thermal radiation. Anuar et al*.*^33^ inspected the impact of radiative hybrid nanofluid and the flow is assumed in a rotating sheet. Tassaddiq et al*.*^34^ calculated the heat and mass transfer phenomena using hybrid nano-liquids and the fluid is assumed in rotating geometry. Ramzan et al*.*^35^ examined the heat transfer using dissimilar shaped nanoparticles for advance cooling process. In this study they considered the fluid flow in a rotating frame.

The research of thermal radiation in modern science has a wide range of industrial and engineering and a variety of thermal applications in industries and cooling systems. Keeping in mind these unique applications related to thermal radiation many research scholars have been considered the influence of thermal radiation in various fluid flow problems and physical situation. In the early days the thermal impacts of radiation on the atmosphere were done by Idso and Jackson^36^. Afterwards, the study of thermal radiation was a hot topic for the researchers due to a large scale of industrial and engineering applications. Gray and Muller^37^ studied some engineering aspects of thermal radiation by calculating radiative heat transfer with applications. Recently, Modest and Mazumder^38^ collected some of the advance applications of thermal radiation with heat transfer in their book. Kumar et al*.*^39^ studied some global advances using solar thermal energy technologies and get some useful energy in solar systems with idea of thermal radiation. This idea was successful and have a lot of energy was restored during this analysis which were used in many energy systems and modern technologies. Shah et al.^40^ investigated the role of thermal radiation with the MHD acting on the fluid and highlight the dynamics of the fluid containing the CNT in domain of stretching sheet. Similarly, Tahir et al*.*^41^, where the authors investigated Techno-economic assessment using thermal radiation applications and power generation systems in energy barriers in various physical systems. The applications of thermal radiation are not only used industrial and engineering problems it has useful applications in medical sciences. Therefore, the researchers Hsu et al.^42^ investigated some useful applications in medical science by highlighting human body radiation and how it causes heating and cooling. According to this research maintaining the body temperature is necessary and basic need for living. Similarly, the researchers Petela^43^, discussed engineering thermodynamics, Navarro et al.^44^ studied thermal radiation in fluid dynamics and Wehinger and Flaischlen^45^ derive the moeling of thermal radiation in computational fluid dynamics all the authors studied the advance and unique applications of thermal radiation in various circumstances.

The study of fractional calculus (FC) attracted the scholars around the world due to its important hidden properties which called as memory. This property of memory is a hidden property in many systems which can be investigated by using FC. The research of FC have widely used in many physical situations from the last few decades. Many mathematicians developed new fractional differential operators for the advance applications and each definition have unique property and applications in different circumstances. The fractional derivatives are Riemann–Liouville, Caputo, Caputo and Fabrizio, Atangana and Baleanu fractional derivatives. In the present analysis we focused to highlight the impact of newly developed fractional operator namely, Constant Proportional Caputo CPC operator. Less results have been provided on this fractional derivatives. We can mention, Gunerhan et al*.*^46^, where the authors investigated CPC fractional operator highlighting its impact on the HIV model. Similarly, Siddique et al*.*^47^ analyzed the impact of CPC operator during the analysis of blood liquor model. Ali et al*.*^48^ developed power law memory on the flow of hybrid nanofluids by applying the CPC fractional operator. Baleanu et al*.*^49^ calculated CPC operator by combining the Caputo and proportional operator and classical differ-integrals. Karatas et al*.*^50^ discussed the CPC operator by applying the Laplace transformation method. Aleem et al*.*^51^ investigated the impact of heat transfer analysis of MHD Casson fluid flow and utilizing CPC fractional operator.

On the bases of the provided literature the present study is targeted on the analysis of the Jeffery tri-hybrid nanofluid past over an infinite vertical plate upward along x-axis in a rotating. In this analysis we have to main outcomes, first in this study we have considered newly developed idea of tri-hybrid nano-liquids with different shape of nanoparticles suspension in a single base fluid. The spherical shape ${\text{CuO}}$, platelet shape ${\text{Al}}_{2} {\text{O}}_{3}$ and cylindrical shape ${\text{TiO}}_{2}$ nanoparticles are added in engine oil to constitute tri-hybrid nanofluid aiming at obtaining better thermal performance. Secondly, in this analysis new definition is obtained by combining the Caputo and proportional derivative and form new fractional operator known as constant proportional Caputo CPC operator. No work is reported to highlight the impact of tri-hybrid nano-liquids on the fluid flow and CPC fractional operator. To fill this gape in the present analysis we focus to evaluate the impact of CPC operator and tri-hybrid nanofluid for advance applications in modern science. The proposed problem is formulated in the form of PDE’s with appropriate IC’s and BC’s. The numerical results obtained by using the Laplace transform technique. Moreover, the parameter of interest is visualized on the flow and heat profiles using MATHCAD software. Finally, the engineering quantities are calculated and presented in tables**.**

## Mathematical modeling

**Figure 1:**
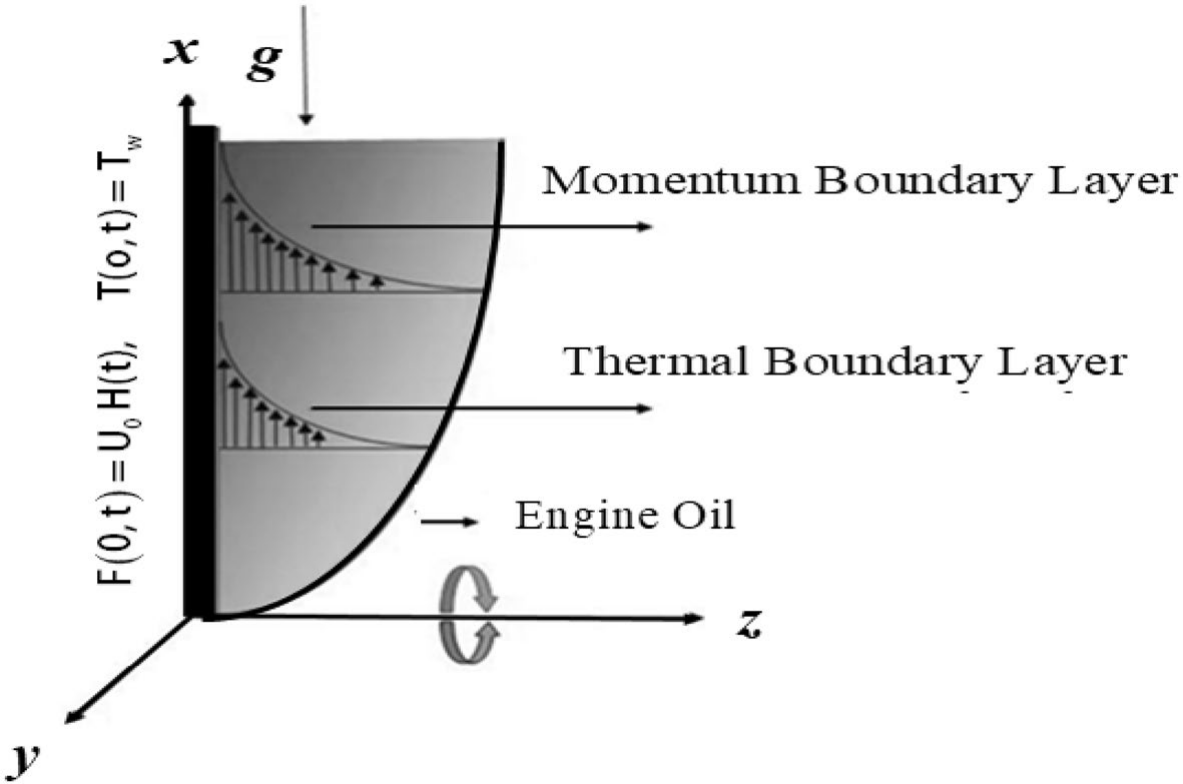
Sketch of the flow regime.

**Figure 2:**
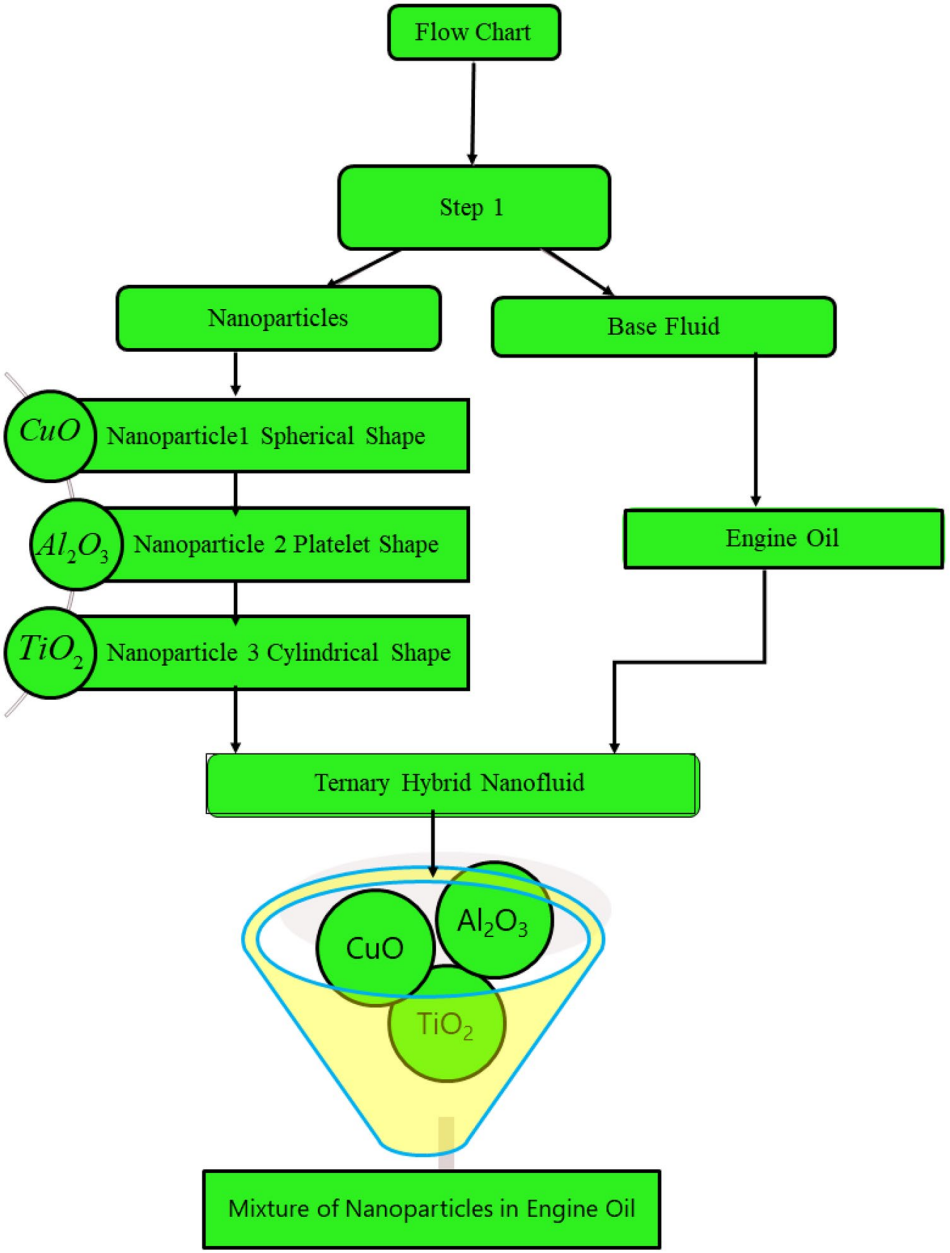
The preparation method for ternary hybrid nanofluid.

Consider Jeffrey tri-hybrid nanofluid past over an infinite vertical plate upward along $x{ - }$ axis in a rotating frame. The engine oil based tri-hybrid nanofluid and the vertical plate is considered as in axisymmetric rotation with the magnitude of angular velocity “$\Omega$” along $z -$* axis*. In this investigation engine oil is taken as base fluid due to its wide applications in daily life. To increase the thermal properties of the engine oil base fluid, three different nanoparticles are suspended, aiming at enhancing the rate of heat transfer. As a result, we obtain tri-hybrid nanofluid, where all nanoparticles are dispersed according to a uniform distribution in the base fluid EO. The fluid lies in $xy -$ plane and occupies the space $z > 0$. The result can be summarized as in Fig. 1. Furthermore, the flow chart for the preparation method of engine oil base tri-nano-liquids is given in Fig. 2.

The Jeffery fluid lying on the plate at rest when time $t = 0,$ and with temperature $T_{\infty }$. Then, taking $t > 0^{ + }$, the plate, externally disturbed, start moving with velocity $U_{0}$, then affecting the fluid near the plate. This triggering event causes the plate temperature to rise to $T_{w} > T_{\infty }$, to then remain unchanged. Since the fluid is considered in a rotating frame, the associated velocity field reads:

1 $$ \overrightarrow {V} = \left\{ {u\left( {z,t} \right),\,v\left( {z,t} \right),\Omega } \right\}, $$

with constitutive fluid equation:

2 $$ \rho_{thnf} \left[ {\frac{dv}{{dt}} + 2\overrightarrow {\Omega } \times \overrightarrow {V} + \overrightarrow {\Omega } \times \left( {\overrightarrow {\Omega } \times \overrightarrow {r} } \right)} \right] = divT + \rho \overrightarrow {b} , $$

where the terms $\rho_{thnf}$, $\overrightarrow {\Omega }$, $T$, $\overrightarrow {r}$ and $\rho \overrightarrow {b}$, represent the density of tri-hybrid nanofluid, the rotation parameter, the Cauchy stress tensor, the position vector in $xy -$ plane and the body forces, respectively.

In particular, the Cauchy stress tensor for Jeffrey fluid can be expressed as:

3 $$ T = - PI + S $$

where $P$ represents pressure, $I$ indicates the identity tensor, while $S$ is given by:

4 $$ S = \frac{{\mu_{thnf} }}{{\left( {1 + \lambda_{1} } \right)}}\left( {1 + \lambda_{2} \frac{d}{dt}} \right)A_{1} , $$

with $\mu_{thnf}$ representing the dynamic viscosity of tri-hybrid nanofluid, while the Jeffrey fluid parameters are denoted by $\lambda_{1}$ and $\lambda_{2}$, and $A_{1}$ stand for the first Rivlin Erickson tensor. In particular, $A_{1}$ is given by

5 $$ A_{1} = L + L^{T} , $$

where

6 $$ L = \nabla \overrightarrow {V} , $$

7 $$ \rho \overrightarrow {b} = \rho \overrightarrow {g} . $$

$\rho \overrightarrow {b} \,\,\,{\text{or}}\,\rho \overrightarrow {g}$ representing the body forces.

Applying the Boussinesq’s approximation^52^, is it possible to derive the governing equations for the Jeffery tri-hybrid nanofluid as follows:

8 $$ \rho_{thnf} \left[ {\frac{\partial u}{{\partial t}} - 2\Omega v} \right] = \frac{{\mu_{thnf} }}{{\left( {1 + \lambda_{1} } \right)}}\left( {1 + \lambda_{2} \frac{d}{dt}} \right)\frac{{\partial^{2} u}}{{\partial z^{2} }} + g\left( {\rho \beta_{T} } \right)_{thnf} \left( {T - T_{\infty } } \right) $$

and

9 $$ \rho_{thnf} \left[ {\frac{\partial v}{{\partial t}} + 2\Omega u} \right] = \frac{{\mu_{thnf} }}{{\left( {1 + \lambda_{1} } \right)}}\left( {1 + \lambda_{2} \frac{d}{dt}} \right)\frac{{\partial^{2} v}}{{\partial z^{2} }}, $$

hence, exploiting both Eq. (8) and Eq. (9), we have:

10 $$ \rho_{thnf} \left[ {\frac{\partial F}{{\partial t}} + 2i\Omega F} \right] = \frac{{\mu_{thnf} }}{{\left( {1 + \lambda_{1} } \right)}}\left( {1 + \lambda_{2} \frac{d}{dt}} \right)\frac{{\partial^{2} F}}{{\partial z^{2} }} + g\left( {\rho \beta_{T} } \right)_{thnf} \left( {T - T_{\infty } } \right). $$

where $F$ represents the so-called complex velocity, being expressed by $F = u + iv$.

The temperature equation with thermal radiation can be expressed as:

11 $$ \left( {\rho C_{p} } \right)_{thnf} \frac{\partial T(z,t)}{{\partial t}} = k_{thnf} \frac{{\partial^{2} T(z,t)}}{{\partial z^{2} }} - \frac{{\partial q_{r} }}{\partial z}, $$

where $q_{r}$ is defined as ^53^,

12 $$ q_{r} = \frac{{4\sigma^{*} }}{{3k_{1}^{*} }}\frac{{\partial T^{4} }}{\partial z}, $$

where $\sigma^{*}$, $k_{1}^{*}$ represents the constant of Stefan-Boltzmann and the coefficient of mean absorption respectively.

Moreover, the temperature during the motion of fluid is considered to be very small. Hence $T^{4}$ can be linearized by using the Taylor expansion around the initial thermal state $T_{\infty }$, obtaining

13 $$ T^{4} = 4T_{\infty }^{3} T - 3T_{\infty }^{4} ......... $$

Neglecting higher order terms in Eq. (13), and by Eq. (12), we can rewrite Eq. (11) as follows:

14 $$ \left( {\rho C_{p} } \right)_{thnf} \frac{\partial T(z,t)}{{\partial t}} = \left[ {k_{thnf} + \frac{{16\sigma^{*} T_{\infty }^{3} }}{{3k_{1}^{*} }}} \right]\frac{{\partial^{2} T(z,t)}}{{\partial z^{2} }}, $$

where $\rho_{thnf}$, $F,\,\,T,\,\,\mu_{thnf} ,\,\,\,\left( {\rho C_{p} } \right)_{thnf} {,} \; \left( {\rho \beta_{T} } \right)_{thnf} \,,\,\,k_{thnf} \,\,{\text{and}}\,{\text{q}}_{r}$ represent the density, the complex velocity, temperature, dynamic viscosity, capacitance, thermal conductivity coefficient, thermal conductivity and radiative heat flux, respectively. Where in the subscript $thnf$ represent the properties of tri-hybrid nano-liquids.

The physical IC’s and BC’s are given as under:

15 $$ \left. \begin{gathered} F\left( {z,t} \right) = 0,\,\,T\left( {z,t} \right) = T_{\infty } ,\,\,{\text{for}}\,\,t = 0 \hfill \\ F\left( {z,t} \right) = U_{0} H\left( t \right),\,\,T\left( {z,t} \right) = T_{w} ,\,\,{\text{for}}\,\,z = 0\,\,{\text{and}}\,\,t > 0 \hfill \\ F\left( {z,t} \right) = 0,\,\,T\left( {z,t} \right) = T_{\infty } ,\,\,{\text{for}}\,\,z \to \infty \,\,{\text{and}}\,\,t > 0 \hfill \\ \end{gathered} \right\} $$

## The evaluation of Tri-Hybrid nanofluid properties:

This section provides a brief description of thermos-physical characteristics of tri-hybrid nano-liquids. This research highlights the performance of various kinds of additives forming a mixture which is called tri-hybrid nano-liquids. In particular, we consider the case when the spherical shape ${\text{CuO}}$, platelet shape ${\text{Al}}_{2} {\text{O}}_{3}$ and cylindrical shape ${\text{TiO}}_{2}$ nano additives are dissolve in engine oil. To express the mixture of these nano additives we use mixture model^58^. The properties of tri-hybrid nanofluid are given as follows:

16 $$ \rho_{thnf} = \left( {1 - \phi_{{{\text{CuO}}}} - \phi_{{{\text{Al}}_{2} {\text{O}}_{3} }} - \phi_{{{\text{TiO}}_{2} }} } \right)\rho_{EO} + \phi_{{{\text{CuO}}}} \rho_{{{\text{CuO}}}} + \phi_{{{\text{Al}}_{2} {\text{O}}_{3} }} \rho_{{{\text{Al}}_{2} {\text{O}}_{3} }} + \phi_{{{\text{TiO}}_{2} }} \rho_{{{\text{TiO}}_{2} }} , $$

17 $$ \begin{gathered} \left( {\rho C_{p} } \right)_{hnf} = \left( {1 - \phi_{{{\text{CuO}}}} - \phi_{{{\text{Al}}_{2} {\text{O}}_{3} }} - \phi_{{{\text{TiO}}_{2} }} } \right)\left( {\rho C_{p} } \right)_{EO} \hfill \\ \quad \quad \quad \quad \; + \phi_{{{\text{CuO}}}} \left( {\rho C_{p} } \right)_{{{\text{CuO}}}} + \phi_{{{\text{Al}}_{2} {\text{O}}_{3} }} \left( {\rho C_{p} } \right)_{{{\text{Al}}_{2} {\text{O}}_{3} }} + \phi_{{TiO_{2} }} \left( {\rho C_{p} } \right)_{{{\text{TiO}}_{2} }} , \hfill \\ \end{gathered} $$

18 $$ \begin{gathered} \left( {\rho \beta_{T} } \right)_{thnf} = \left( {1 - \phi_{{{\text{CuO}}}} - \phi_{{{\text{Al}}_{2} {\text{O}}_{3} }} - \phi_{{TiO_{2} }} } \right)\left( {\rho \beta_{T} } \right)_{EO} \hfill \\ \quad \quad \quad \quad \; + \phi_{{{\text{CuO}}}} \left( {\rho \beta_{T} } \right)_{{{\text{CuO}}}} + \phi_{{{\text{Al}}_{2} {\text{O}}_{3} }} \left( {\rho \beta_{T} } \right)_{{{\text{Al}}_{2} {\text{O}}_{3} }} + \phi_{{{\text{TiO}}_{2} }} \left( {\rho \beta_{T} } \right)_{{{\text{TiO}}_{2} }} . \hfill \\ \end{gathered} $$

According to Maxwell model^59^, thermal conductivity of tri-hybrid nano-liquids can be expressed as:

19 $$ \frac{{k_{nf} }}{{k_{bf} }} = \frac{{k_{1} + (n - 1)k_{bf} + (n - 1)\phi (k_{1} - k_{bf} )}}{{k_{1} + (n - 1)k_{bf} - (k_{1} - k_{bf} )}} $$

From the expression given above $\Psi$ represents the sphericity and $n = \left( {\frac{3}{\Psi }} \right)$ shows the shape factor of the nanoparticles.

Further this research explained the three various kinds of additives with different shapes have been calculated. First, we consider the values of $\Psi = 1$ and $n = 1$ the expression then obtained for the spherical shape. Secondly, by considering the values of $\Psi = 0.612$ and $n = 4.9$ then the expression will used for cylinder shape of the nanoparticle. Finally, if we consider $\Psi = 0.52$ and $n = 5.7$ the result will then obtain for platelet shape of nanoparticles.

Moreover, by obtaining the expressions for viscosity and thermal conductivity for various shape additives of viscosity using Mixture model^58^, we have:

20 $$ \left. \begin{gathered} \frac{{\mu_{{{\text{CuO}}}} }}{{\mu_{EO} }} = 1 + 2.5\phi + 6.2\phi^{2} \hfill \\ \frac{{k_{{{\text{CuO}}}} }}{{k_{EO} }} = \frac{{k_{{{\text{CuO}}}} + 2k_{EO} + 2\phi (k_{{{\text{CuO}}}} - k_{EO} )}}{{k_{{{\text{CuO}}}} + 2k_{EO} - \phi (k_{{{\text{CuO}}}} - k_{EO} )}} \hfill \\ \end{gathered} \right\} \mapsto \quad \left( {{\text{nanoparticle}} - {\text{1 spherical shaped}}} \right) $$

21 $$ \left. \begin{gathered} \frac{{\mu_{{{\text{Al}}_{2} {\text{O}}_{3} }} }}{{\mu_{EO} }} = 1 + 13.5\phi + 904.4\phi^{2} \hfill \\ \frac{{k_{{{\text{Al}}_{2} {\text{O}}_{3} }} }}{{k_{EO} }} = \frac{{k_{{{\text{Al}}_{2} {\text{O}}_{3} }} + 3.9k_{EO} + 3.9\phi (k_{{{\text{Al}}_{2} {\text{O}}_{3} }} - k_{EO} )}}{{k_{{{\text{Al}}_{2} {\text{O}}_{3} }} + 3.9k_{EO} - \phi (k_{{{\text{Al}}_{2} {\text{O}}_{3} }} - k_{EO} )}} \hfill \\ \end{gathered} \right\} \mapsto \quad \left( {{\text{nanoparticle}} - {\text{2 cylindrical shaped}}} \right) $$

22 $$ \left. \begin{gathered} \frac{{\mu_{{{\text{TiO}}_{2} }} }}{{\mu_{EO} }} = 1 + 37.1\phi + 612.6\phi^{2} \hfill \\ \frac{{k_{{{\text{TiO}}_{2} }} }}{{k_{EO} }} = \frac{{k_{{{\text{TiO}}_{2} }} + 4.7k_{EO} + 4.7\phi (k_{{{\text{TiO}}_{2} }} - k_{EO} )}}{{k_{{{\text{TiO}}_{2} }} + 4.7k_{EO} - \phi (k_{{{\text{TiO}}_{2} }} - k_{EO} )}} \hfill \\ \end{gathered} \right\} \mapsto \quad \left( {{\text{nanoparticle}} - {\text{3 platelet shaped}}} \right) $$

From the Eqs. (20)–(22) the effective dynamic viscosity for tri-hybrid nano additives can be expressed as follow:

23 $$ \mu_{thnf} = \frac{{\mu_{{{\text{CuO}}}} \phi_{{{\text{CuO}}}} + \mu_{{{\text{Al}}_{2} {\text{O}}_{3} }} \phi_{{{\text{Al}}_{2} {\text{O}}_{3} }} + \mu_{{{\text{TiO}}_{2} }} \phi_{{{\text{TiO}}_{2} }} }}{{\phi_{thnf} }}. $$

with thermal conductivity, for each different shaped nanoparticle, given by:

24 $$ k_{thnf} = \frac{{k_{{{\text{CuO}}}} \phi_{{{\text{CuO}}}} + k_{{{\text{Al}}_{{2}} {\text{O}}_{3} }} \phi_{{{\text{Al}}_{2} {\text{O}}_{3} }} + k_{{{\text{TiO}}_{2} }} \phi_{{{\text{TiO}}_{2} }} }}{{\phi_{thnf} }} $$

where $$\phi_{thnf} = \phi_{{{\text{CuO}}}} + \phi_{{{\text{Al}}_{2} {\text{O}}_{3} }} + \phi_{{{\text{TiO}}_{2} }}$$.

Furthermore, in the given analysis it can be noticed that the spherical shape ${\text{CuO}}$, the platelet shape ${\text{Al}}_{2} {\text{O}}_{3}$ and the cylindrical shape ${\text{TiO}}_{2}$ nano additives are added in same quantity in engine oil:

Then, applying the Buckingham-Pi theorem, we obtain the following dimensional variables:

25 $$ F^{*} = \frac{F}{{U_{0} }},\,\,\,z^{*} = \frac{{U_{0} }}{\upsilon }z,\,\,\,\,t^{*} = \frac{{U_{0}^{2} }}{\upsilon }t,\,\,\theta = \frac{{T - T_{\infty } }}{{T_{w} - T_{\infty } }}. $$

which allow to derive the associated dimensionless system as follows:

26 $$ \frac{{\partial F\left( {z,t} \right)}}{\partial t} + 2irF\left( {z,t} \right) = \frac{{m_{1} }}{{\left( {1 + \lambda_{1} } \right)}}\left( {1 + \lambda \frac{\partial }{\partial t}} \right)\frac{{\partial^{2} F\left( {z,t} \right)}}{{\partial z^{2} }} + m_{2} Gr\theta \left( {z,t} \right) $$

27 $$ \frac{{\partial \theta \left( {z,t} \right)}}{\partial t} = \frac{1}{\Pr }\left( {\frac{{Nr + a_{4} }}{{a_{3} }}} \right)\frac{{\partial^{2} \theta \left( {z,t} \right)}}{{\partial z^{2} }}, $$

accordingly, initial and boundary conditions read:

28 $$ \left. \begin{gathered} F\left( {z,t} \right) = 0,\,\,T\left( {z,t} \right) = 0,\,\,\,\,\,{\text{for}}\,\,t = 0 \hfill \\ F\left( {z,t} \right) = 1,\,\,\,\,T\left( {z,t} \right) = 1,\,\,\,\,{\text{for}}\,\,z = 0\,\,{\text{and}}\,\,t > 0 \hfill \\ F\left( {z,t} \right) = 0,\,\,T\left( {z,t} \right) = 0,\,\,\,\,\,{\text{for}}\,\,z \to \infty \,\,{\text{and}}\,\,t > 0 \hfill \\ \end{gathered} \right\} $$

During the demineralization process we get the following parameters:

$$ r = \frac{\Omega \upsilon }{{U_{0}^{2} }},\,\,\,\lambda = \frac{{\lambda_{2} U_{0}^{2} }}{\upsilon },\,\,Gr = \frac{{g\beta_{T} \upsilon \left( {T_{w} - T_{\infty } } \right)}}{{U_{0}^{3} }},\,Nr = \frac{{16\sigma^{*} T_{\infty }^{3} }}{{3k_{1}^{*} k_{f} }},\,\,\Pr = \frac{{\mu C_{p} }}{k} $$

$$ a_{0} = \left( {1 - \phi_{{{\text{CuO}}}} - \phi_{{{\text{Al}}_{2} {\text{O}}_{3} }} - \phi_{{{\text{TiO}}_{2} }} } \right) + \frac{{\phi_{{{\text{CuO}}}} \rho_{{{\text{CuO}}}} }}{{\rho_{EO} }} + \frac{{\phi_{{{\text{Al}}_{2} {\text{O}}_{3} }} \rho_{{{\text{Al}}_{2} {\text{O}}_{3} }} }}{{\rho_{EO} }} + \frac{{\phi_{{{\text{TiO}}_{2} }} \rho_{{{\text{TiO}}_{2} }} }}{{\rho_{EO} }},\,\,\,a_{1} = \frac{{b_{1} \phi_{{{\text{CuO}}}} + b_{2} \phi_{{{\text{Al}}_{2} {\text{O}}_{3} }} + b_{3} \phi_{{{\text{TiO}}_{2} }} }}{{\phi_{hnf} }}, $$

$$ a_{2} = \left( {1 - \phi_{{{\text{CuO}}}} - \phi_{{{\text{Al}}_{2} {\text{O}}_{3} }} - \phi_{{{\text{TiO}}_{2} }} } \right) + \frac{{\phi_{{{\text{CuO}}}} \left( {\rho \beta_{T} } \right)_{{{\text{CuO}}}} }}{{\left( {\rho \beta_{T} } \right)_{EO} }} + \frac{{\phi_{{{\text{Al}}_{2} {\text{O}}_{3} }} \left( {\rho \beta_{T} } \right)_{{{\text{Al}}_{2} {\text{O}}_{3} }} }}{{\left( {\rho \beta_{T} } \right)_{EO} }} + \frac{{\phi_{{{\text{TiO}}_{2} }} \left( {\rho \beta_{T} } \right)_{{{\text{TiO}}_{2} }} }}{{\left( {\rho \beta_{T} } \right)_{EO} }}, \, $$

$$ a_{3} = \left( {1 - \phi_{{{\text{CuO}}}} - \phi_{{{\text{Al}}_{2} {\text{O}}_{3} }} - \phi_{{{\text{TiO}}_{2} }} } \right) + \frac{{\phi_{{{\text{CuO}}}} \left( {\rho C_{p} } \right)_{{{\text{CuO}}}} }}{{\left( {\rho C_{p} } \right)_{EO} }} + \frac{{\phi_{{{\text{Al}}_{2} {\text{O}}_{3} }} \left( {\rho C_{p} } \right)_{{{\text{Al}}_{2} {\text{O}}_{3} }} }}{{\left( {\rho C_{p} } \right)_{EO} }} + \frac{{\phi_{{{\text{TiO}}_{2} }} \left( {\rho C_{p} } \right)_{{{\text{TiO}}_{2} }} }}{{\left( {\rho C_{p} } \right)_{EO} }}, $$

$$a_{4} = \frac{{b_{4} \phi_{{{\text{CuO}}}} + b_{5} \phi_{{{\text{Al}}_{2} {\text{O}}_{3} }} + b_{6} \phi_{{{\text{TiO}}_{2} }} }}{{\phi_{hnf} }}$$,$$m_{1} = \frac{{a_{1} }}{{a_{0} }}$$ ,$$m_{2} = \frac{{a_{2} }}{{a_{0} }}$$ ,

$$ b_{1} = 1 + 2.5\phi + 6.2\phi^{2} ,\,\,\,\,b_{2} = 1 + 13.5\phi + 904.4\phi^{2} , $$

$$b_{3} = 1 + 37.1\phi + 612.6\phi^{2} ,\,\,\,b_{4} = \frac{{k_{{{\text{CuO}}}} + 2k_{EO} + 2\phi (k_{{{\text{CuO}}}} - k_{EO} )}}{{k_{{{\text{CuO}}}} + 2k_{EO} - (k_{{{\text{CuO}}}} - k_{EO} )}}$$,

$$b_{5} = \frac{{k_{{{\text{Al}}_{2} {\text{O}}_{3} }} + 3.9k_{EO} + 3.9\phi (k_{{{\text{Al}}_{2} {\text{O}}_{3} }} - k_{EO} )}}{{k_{{{\text{Al}}_{2} {\text{O}}_{3} }} + 3.9k_{EO} - (k_{{{\text{Al}}_{2} {\text{O}}_{3} }} - k_{EO} )}},\,\,\,\,\,\,b_{6} = \frac{{k_{{{\text{TiO}}_{2} }} + 4.7k_{EO} + 4.7\phi (k_{{{\text{TiO}}_{2} }} - k_{EO} )}}{{k_{{{\text{TiO}}_{2} }} + 4.7k_{EO} - (k_{{TiO_{2} }} - k_{EO} )}}$$.

## Methodology

**Figure 3:**
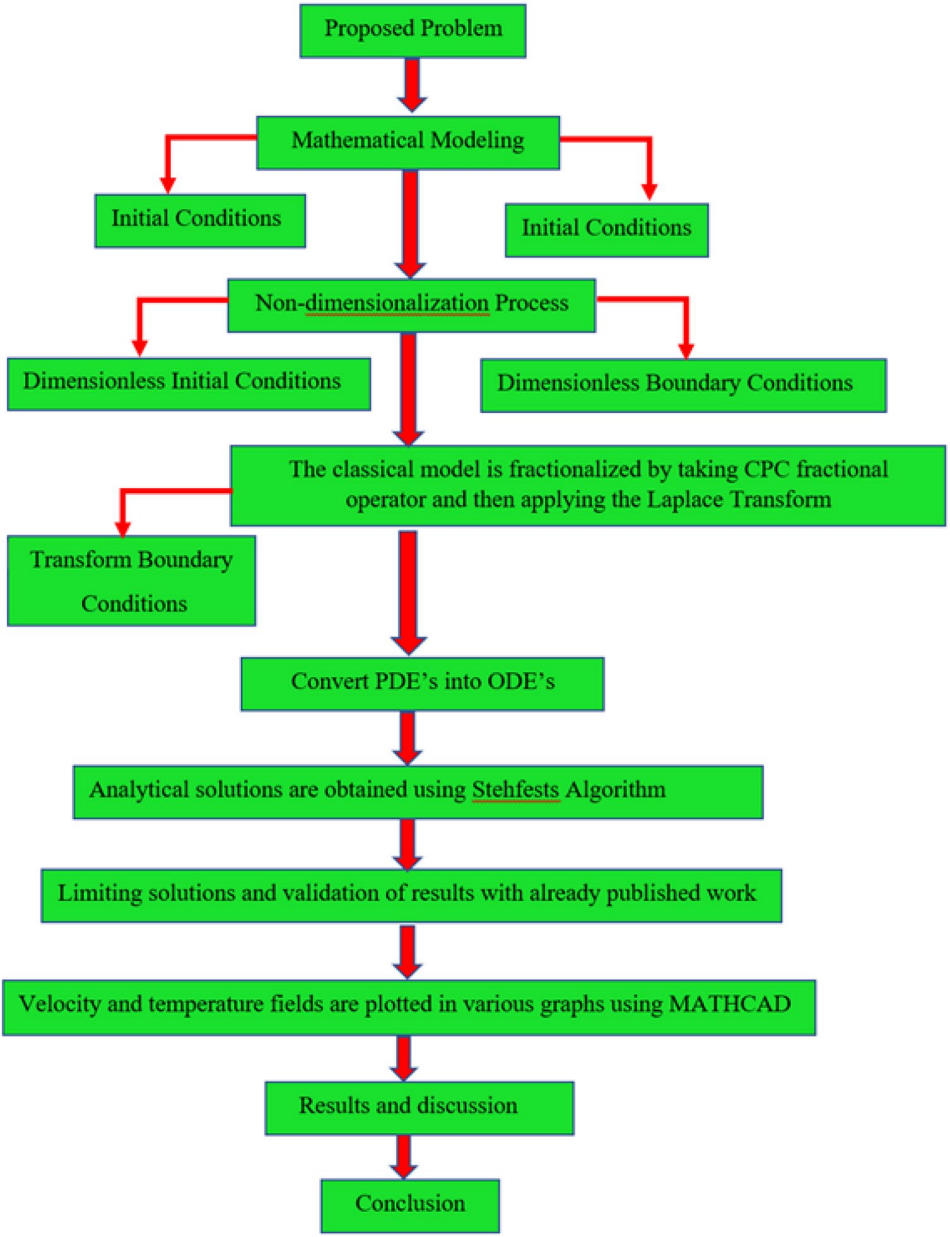
Operational Framework.

This section provides solution methodology of the present research. The flow chart of the present article can be define as, first we propose a model with physical IC’s and BC’s. then the considered system of equations can be transformed by applying the dimensionless variables. After depersonalization the classical model is fractionalized by taking CPC operator. The analytical solutions have been obtained by using the applications of the Laplace transform. The obtained system of equations are complex and not an easy task to get the exact solutions. Therefore, to invert expressions we derived via the Laplace transform, several methods can be used, but we opted for the Stehfests algorithm ref^50^. (see Eq. 49) using MATHCAD software the detail of which is given at the end of section "Solutions With Constant Proportional Caputo Operator". Furthermore, for plotting the graphs of the present solutions we have used MATHCAD 15 which can be found in the link https://mathcad.software.informer.com/15.0/. In addition to this the detailed methodology of the present research is provided in the flow chart which is given in Fig. 3.

## Basics on Caputo approach

In view of next computations, let us recall the Caputo fractional derivative definition, see^60^:

29 $$^{C}_{0} D_{t}^{\alpha } f(t) = \frac{1}{\Gamma (1 - \alpha )}\int_{0}^{t} {f^{^{\prime}} \left( \tau \right)\left( {t - \tau } \right)^{ - \alpha } d\tau .} $$

$f(t)$ being a differentiable function, while $\alpha \in (0,1)$ represents the order of the fractional derivative, at initial time $t = 0,$

It is worth recalling that the Caputo derivative extends the Riemann–Liouville integral:

30 $$^{RL}_{0} I_{t}^{\beta } f(t) = \frac{1}{\Gamma (\beta )}\int_{0}^{t} {f\left( \tau \right)\left( {t - \tau } \right)^{\beta - 1} d\tau ,} $$

Furthermore, see^61^. we also recall the general, non-fractional, differential operator known as proportional, or conformable, given by:

31 $$^{P} D_{\alpha } f(t) = N_{1} (\alpha ,t)f(t) + N_{0} (\alpha ,t)f^{^{\prime}} (t), $$

where $$N_{0}$$ and $$N_{1}$$ are the functions of $$t\,\,{\text{and}}\,\,\alpha$$, $$\alpha \in [0,1],[0,1]$$$$,$$ verifying the below conditions for all real values of $$t:$$

32 $$ \mathop {\lim }\limits_{{\alpha \to 0^{ + } }} N_{0} (\alpha ,t) = 0;\quad \quad \mathop {\lim }\limits_{{\alpha \to 1^{ - } }} N_{0} (\alpha ,t) = 1,N_{0} (\alpha ,t) \ne 0,\;{\text{for}}\,{\text{all}}\,\,\,\,\,\,\alpha \in (0,1], $$

and

33 $$ \mathop {\lim }\limits_{{\alpha \to 0^{ + } }} N_{1} (\alpha ,t) = 1;\quad \mathop {\lim }\limits_{{\alpha \to 1^{ - } }} N_{1} (\alpha ,t) = 0,\,\,N_{1} (\alpha ,t) \ne 0,\quad {\text{for}}\,{\text{all}}\,\,\,\,\,\,\alpha \in [0,1). $$

The special case of the above functions $N_{0}$ and $N_{1}$ are given by choosing taking $t$ constant, above functions $N_{0}$ and $N_{1}$ only depend on $\alpha$, and we recover the so-called CP “Constant Proportional” (CP) case, represented by:

34 $$^{CP} D_{\alpha } f(t) = N_{1} (\alpha )f(t) + N_{0} (\alpha )f^{^{\prime}} (t). $$

### Model the definition of CPC

The main motivation of the present work rely in considering the recently proposed definition of (CPC) operator. This is a hybrid fractional parameter obtained by combining the CP in Eq. (34) with the standard Caputo operator defined in Eq. (29). The resulting CPC operator, see^49^, is then given by:

35 $$^{CPC}_{0} D_{t}^{\alpha } f(t) = \frac{1}{\Gamma (1 - \alpha )}\int_{0}^{t} {\left[ {N_{1} (\alpha )f(t) + N_{0} (\alpha )f^{^{\prime}} (t)} \right]\left( {t - \tau } \right)^{ - \alpha } d\tau .} $$

with associated Laplace transform:

36 $$ L\left[ {^{CPC}_{0} D_{t}^{\alpha } f(t)} \right] = \left[ {\frac{{N_{1} (\alpha )}}{s} + N_{0} (\alpha )} \right]s^{\alpha } \overline{f} (s) - N_{0} (\alpha )s^{\alpha - 1} f(0), $$

Expression in Eq. (36), can be rewritten as

37 $$ L\left[ {^{CPC}_{0} D_{t}^{\alpha } f(t)} \right] = \left[ {s + \frac{{N_{1} (\alpha )}}{{N_{0} (\alpha )}}} \right]N_{0} (\alpha )s^{\alpha - 1} \overline{f} (s) $$

where $f(t)$ is a differential function, moreover both $f$ and $f^{^{\prime}}$ are locally $L^{1}$ on R^ + and we gain existence for the Laplace transform of $f(t)$, see^49^, for more details.

## Solutions with constant proportional Caputo operator

Applying the CPC operator defined in Eq. (35) to the classical Jeffery model we get:

38 $$^{CPC} D_{t}^{\alpha } F\left( {z,t} \right) + 2irF\left( {z,t} \right) = \frac{{m_{1} }}{{\left( {1 + \lambda_{1} } \right)}}\left( {1 + \lambda^{CPC} D_{t}^{\alpha } } \right)\frac{{\partial^{2} F\left( {z,t} \right)}}{{\partial z^{2} }} + m_{2} Gr\theta \left( {z,t} \right) $$

39 $$^{CPC} D_{t}^{\alpha } \theta \left( {z,t} \right) = \frac{1}{\Pr }\left( {\frac{{Nr + a_{4} }}{{a_{3} }}} \right)\frac{{\partial^{2} \theta \left( {z,t} \right)}}{{\partial z^{2} }}, $$

### Solutions of energy equation with constant proportional Caputo operator

The present problem can be solved numerically using the Laplace transform technique. Therefore, by applying the Laplace transform to Eq. (22), we get:

40 $$ \left[ {\frac{{N_{1} (\alpha )}}{s} + N_{0} (\alpha )} \right]s^{\alpha } \overline{\theta } \left( {z,s} \right) - N_{0} (\alpha )s^{\alpha - 1} \theta \left( {z,0} \right) = \frac{1}{\Pr }\left( {\frac{{Nr + a_{4} }}{{a_{3} }}} \right)\frac{{\partial^{2} \overline{\theta } \left( {z,t} \right)}}{{\partial z^{2} }}, $$

Then, by exploiting initial conditions from Eq. (11) and re-arranging terms in Eq. (40), we can rewrite Eq. (40) as follows:

41 $$ \left[ {s + \frac{{N_{1} (\alpha )}}{{N_{0} (\alpha )}}} \right]N_{0} (\alpha )s^{\alpha - 1} \overline{\theta } \left( {z,s} \right) = \frac{1}{\Pr }\left( {\frac{{Nr + a_{4} }}{{a_{3} }}} \right)\frac{{\partial^{2} \overline{\theta } }}{{\partial z^{2} }}, $$

Accordingly, the result obtained in Eq. (25), can be more effectively written as:

42 $$ \frac{{\partial^{2} \overline{\theta } \left( {z,s} \right)}}{{\partial z^{2} }} - s^{\alpha - 1} \left( {s + \frac{{N_{1} (\alpha )}}{{N_{0} (\alpha )}}} \right)\left( {\frac{{\Pr \cdot N_{0} \left( \alpha \right)a_{3} }}{{Nr + a_{4} }}} \right)\overline{\theta } \left( {z,s} \right) = 0 $$

With solution given by:

43 $$ \overline{\theta } (z,s) = \frac{1}{s}\exp \left( { - z\sqrt {s^{\alpha - 1} \left( {s + \frac{{N_{1} (\alpha )}}{{N_{0} (\alpha )}}} \right)\left( {\frac{{\Pr .N_{0} \left( \alpha \right)a_{3} }}{{Nr + a_{4} }}} \right)} } \right). $$

### Solutions of momentum equation with constant proportional Caputo operator

Analogously, we apply the Laplace transform to Eq. (21), obtaining:

44 $$ \begin{gathered} s^{\alpha - 1} N_{0} (\alpha )\left( {s + \frac{{N_{1} (\alpha )}}{{N_{0} (\alpha )}}} \right)\overline{F} \left( {z,s} \right) + 2ir\overline{F} \left( {z,s} \right) = \hfill \\ \quad \quad \frac{{m_{1} }}{{\left( {1 + \lambda_{1} } \right)}}\left( {1 + \lambda s^{\alpha - 1} N_{0} (\alpha )\left( {s + \frac{{N_{1} (\alpha )}}{{N_{0} (\alpha )}}} \right)} \right)\frac{{\partial^{2} \overline{F} \left( {z,s} \right)}}{{\partial z^{2} }} + m_{2} Gr\overline{\theta } \left( {z,s} \right) \hfill \\ \end{gathered} $$

which can be rewritten as:

45 $$ \begin{gathered} \frac{{\partial^{2} \overline{F} \left( {z,s} \right)}}{{\partial z^{2} }} - \left[ {\frac{{s^{\alpha - 1} N_{0} (\alpha )\left( {s + \frac{{N_{1} (\alpha )}}{{N_{0} (\alpha )}}} \right) + 2ir}}{{\frac{{m_{1} }}{{\left( {1 + \lambda_{1} } \right)}}\left( {1 + \lambda s^{\alpha - 1} N_{0} (\alpha )\left( {s + \frac{{N_{1} (\alpha )}}{{N_{0} (\alpha )}}} \right)} \right)}}} \right]\overline{F} \left( {z,s} \right) = \hfill \\ \quad \quad \quad \quad - \frac{{m_{2} Gr}}{{\frac{{m_{1} }}{{\left( {1 + \lambda_{1} } \right)}}\left( {1 + \lambda s^{\alpha - 1} N_{0} (\alpha )\left( {s + \frac{{N_{1} (\alpha )}}{{N_{0} (\alpha )}}} \right)} \right)}}\overline{\theta } \left( {z,s} \right) \hfill \\ \end{gathered} $$

46 $$ \begin{gathered} \frac{{\partial^{2} \overline{F} \left( {z,s} \right)}}{{\partial z^{2} }} - \left[ {\frac{{s^{\alpha - 1} N_{0} (\alpha )\left( {s + \frac{{N_{1} (\alpha )}}{{N_{0} (\alpha )}}} \right) + 2ir}}{{\frac{{m_{1} }}{{\left( {1 + \lambda_{1} } \right)}}\left( {1 + \lambda s^{\alpha - 1} N_{0} (\alpha )\left( {s + \frac{{N_{1} (\alpha )}}{{N_{0} (\alpha )}}} \right)} \right)}}} \right]\overline{F} \left( {z,s} \right) = \hfill \\ \quad \quad \quad \quad - \frac{{m_{2} Gr}}{{\frac{{m_{1} }}{{\left( {1 + \lambda_{1} } \right)}}\left( {1 + \lambda s^{\alpha - 1} N_{0} (\alpha )\left( {s + \frac{{N_{1} (\alpha )}}{{N_{0} (\alpha )}}} \right)} \right)}}\frac{1}{s}\exp \left( { - z\sqrt {s^{\alpha - 1} \left( {s + \frac{{N_{1} (\alpha )}}{{N_{0} (\alpha )}}} \right)\left( {\frac{{\Pr .N_{0} \left( \alpha \right)a_{3} }}{{Nr + a_{4} }}} \right)} } \right) \hfill \\ \end{gathered} $$

allowing to find:

47 $$ \frac{{\partial^{2} \overline{F} \left( {z,s} \right)}}{{\partial z^{2} }} - X_{1} (s)\overline{F} \left( {z,s} \right) = - X_{2} (s)\frac{1}{s}\exp \left( { - z\sqrt {X_{3} (s)} } \right) $$

And, thanks to the given IC’s and BC’s, the solution of Eq. (47), reads as:

48 $$ \overline{F} \left( {z,s} \right) = \left[ {\frac{1}{s} + \frac{{X_{2} (s)}}{{s\left\{ {X_{3} (s) - X_{1} (s)} \right\}}}} \right]\exp \left( { - z\sqrt {X_{1} (s)} } \right) - \frac{{X_{2} (s)}}{{s\left\{ {X_{3} (s) - X_{1} (s)} \right\}}}\exp \left( { - z\sqrt {X_{3} (s)} } \right) $$

where:

$$X_{1} (s) = \frac{{s^{\alpha - 1} N_{0} (\alpha )\left( {s + \frac{{N_{1} (\alpha )}}{{N_{0} (\alpha )}}} \right) + 2ir}}{{\frac{{m_{1} }}{{\left( {1 + \lambda_{1} } \right)}}\left( {1 + \lambda s^{\alpha - 1} N_{0} (\alpha )\left( {s + \frac{{N_{1} (\alpha )}}{{N_{0} (\alpha )}}} \right)} \right)}}$$ ,

$$X_{2} (s)\frac{{m_{2} Gr}}{{\frac{{m_{1} }}{{\left( {1 + \lambda_{1} } \right)}}\left( {1 + \lambda s^{\alpha - 1} N_{0} (\alpha )\left( {s + \frac{{N_{1} (\alpha )}}{{N_{0} (\alpha )}}} \right)} \right)}}$$ ,

$$X_{3} (s) = s^{\alpha - 1} \left( {s + \frac{{N_{1} (\alpha )}}{{N_{0} (\alpha )}}} \right)\left( {\frac{{\Pr \cdot N_{0} \left( \alpha \right)a_{3} }}{{Nr + a_{4} }}} \right)$$ .

The analytical results are obtained in Eq. (43) and Eq. (48) for the temperature and velocity distributions using the Laplace transform technique. As to invert expressions we derived via the Laplace transform, several methods can be used, see, e.g.,^62^ and^63^. We opted for the Stehfests algorithm, see^64^, obtaining:

49 $$ v(r,\tau ) = \frac{{e^{4.7} }}{\tau }\left[ {\frac{1}{2}v\left( {r,\frac{4.7}{\tau }} \right) + {\text{Re}} \left\{ {\sum\limits_{k = 1}^{{N_{1} }} {\left( { - 1} \right)^{k} } v\left( {r,\frac{4.7 + k\pi i}{\tau }} \right)} \right\}.} \right] $$

where ${\text{Re}} (.)$ is the real part,$i$ stands for the imaginary part, and $N_{1}$ is a natural number indicating the approximation grade.

## Limiting case

This section provide the limitng case by putitng the rotation parameter $r = 0,$ fractional parameter $\alpha \to 1$ and volume fraction parameter of tri-hybrid nanofluid $\phi_{hnf} = 0$ then our fractional tri-hybrid nanofluid model of Jeffery fluid in a rotating frame reduced to the solution classical solution of Jeffery fluid in the absence of rotation which wes done by Khan et al*.*^65^ which verify our botianed soluitons. The comaprsion of our solution to the solutions obtaianed by Khan et al*.*^65^ is elucidated in Fig. 11. This comparison of our solutions to the already published work validate our results.

## Results and discussion

In what follows we recap results and main considerations to better cast the impact of tri-hybrid nanofluid on the generalized Jeffery fluid flowing in a rotating frame. In particular, we considered the suspension of varoius nanoparticles with different shaped in the base fluid engine oil for advance cooling systems. The classical model of Jeffery fluid have been transformed to a fractional model by emplyoing the newly developed CPC operator. The latter has been obtained as the combination of constant prportional and Caputo operator. This allows us to get new insights about the novel employment of tri-hybrid nanofluid in base fluid engine oil. Secondly, the CPC operator has been applied to further analyze the classical model of Jeffery fluid to gain its generalization to its tri-hybrid nanofluid in a rotating frame. Because of lack of explicit solutions for the equations treated in analysing proposed models, we aplied the Laplace transfrom on the system of equations to then numerically solve them by the Stehfests algorithm^64^, the implementing some MATHCAD routines to illustrate them graphically. All the parameters and related impact on the flow and heat have been visualized accordingly. Namely, the impact of volume $\phi_{thnf}$ of ternary hybrid nanofluid $r,\,\,\lambda ,\,\,Gr,\,\,\Pr \,\,{\text{and}}\,\,Nr$, being, respectively, the rotation parameter, the material parameter in dimensionless form, the Grashof number, the Prandtl number and the radiation parameter are highlighted on the flow and heat profiles.

**Figure 4:**
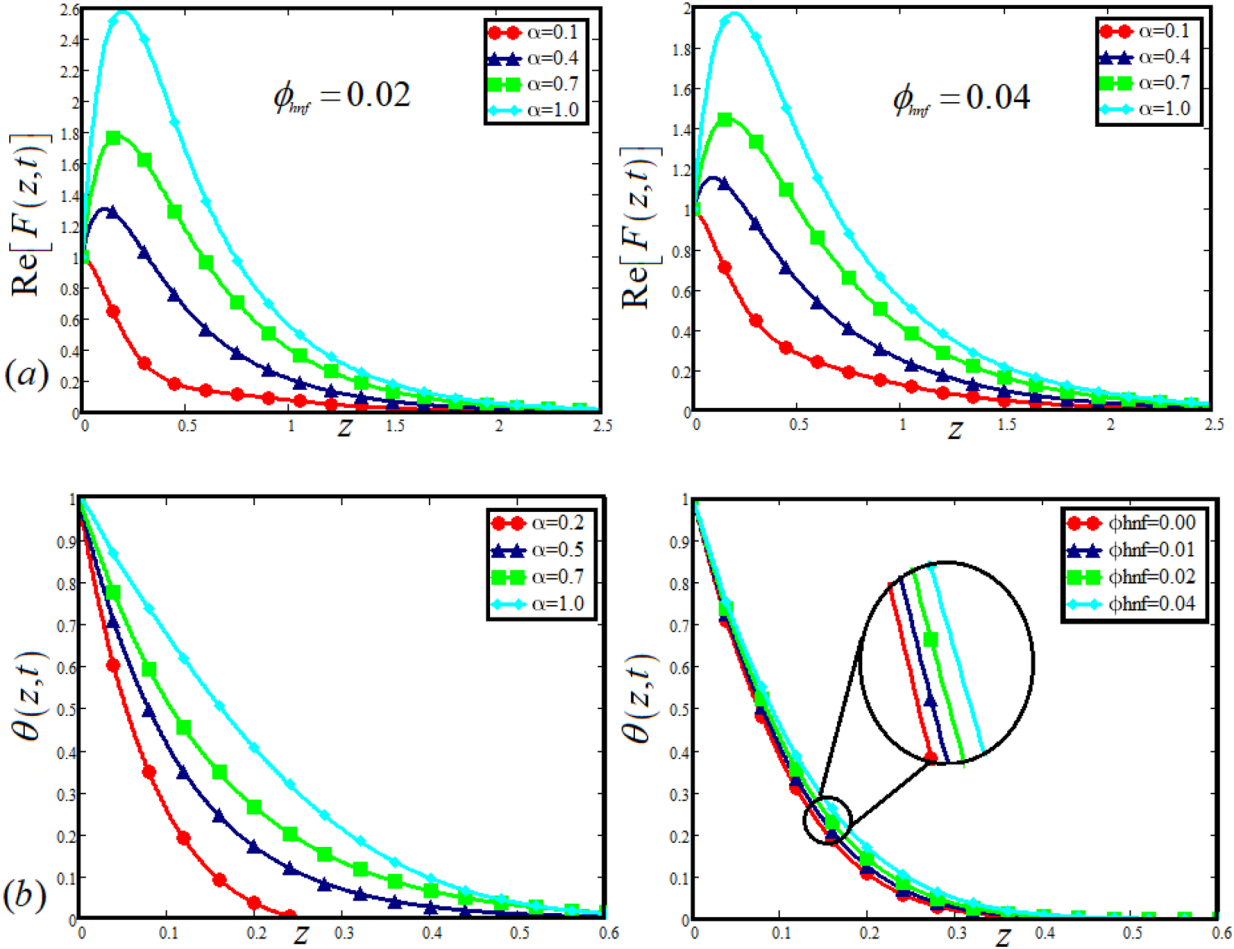
(**a**) The impact of $\alpha$ and $\phi_{hnf}$ on the Jeffery hybrid nanofluid velocity and. (**b**) The impact of $\alpha$ and $\phi_{hnf}$ on the temperature of Jeffery hybrid nanofluid, when $Gr = 80$, $\Pr = 600$, $Nr = 7$, $t = 2.2$, $\lambda = 0.5$, $\lambda_{1} = 2.5$ and $r = 1$.

**Figure 5:**
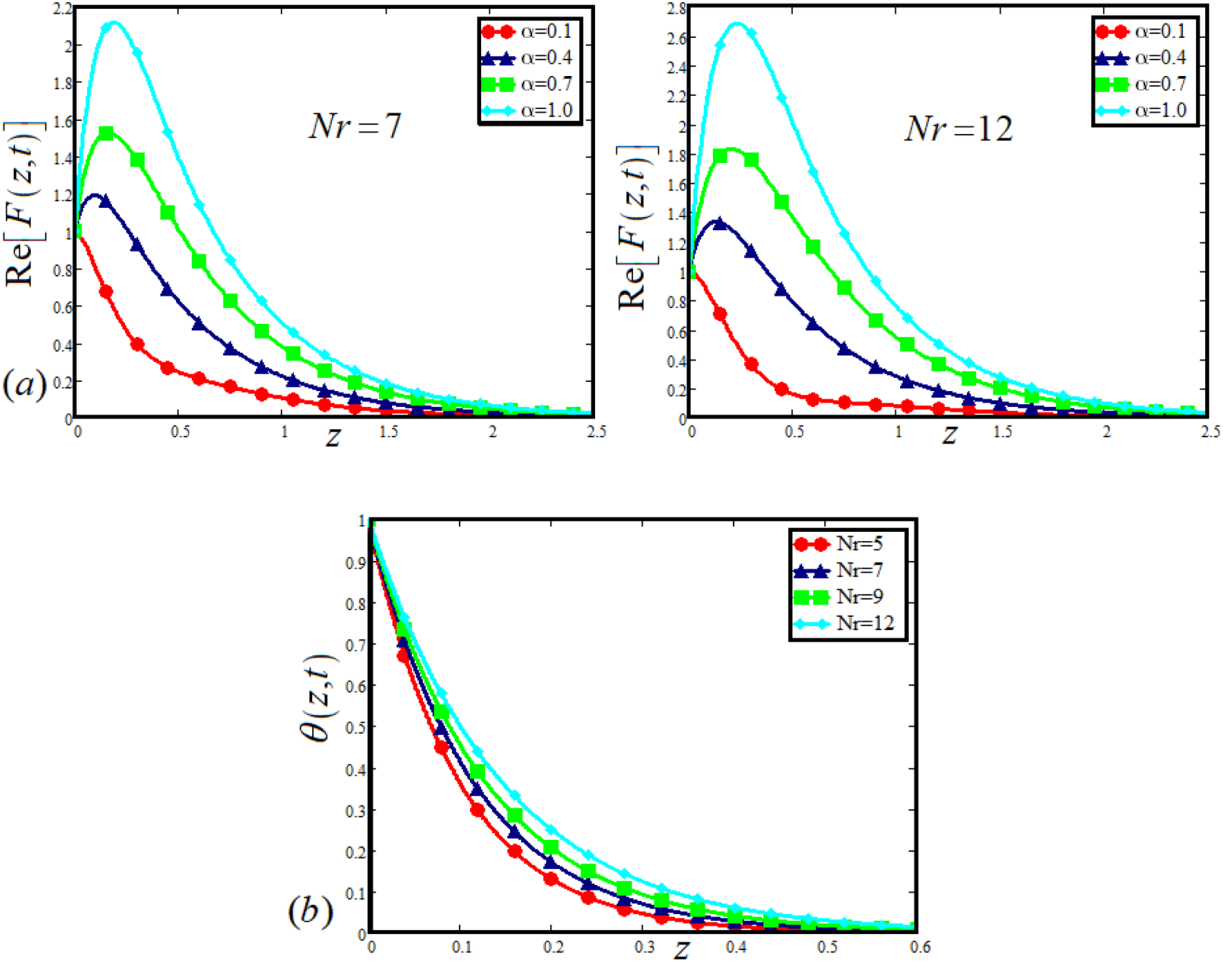
(**a**) The impact of $\alpha$ and $Nr$ on the Jeffery hybrid nanofluid velocity, (**b**) the impact of *α* on the temperature of Jeffery hybrid nanofluid, when $Gr = 80$, $\Pr = 600$, $\phi_{hnf} = 0.03$, $t = 2$ , $\lambda = 0.5$, $\lambda_{1} = 2.5$ and $r = 1$.

**Figure 6:**
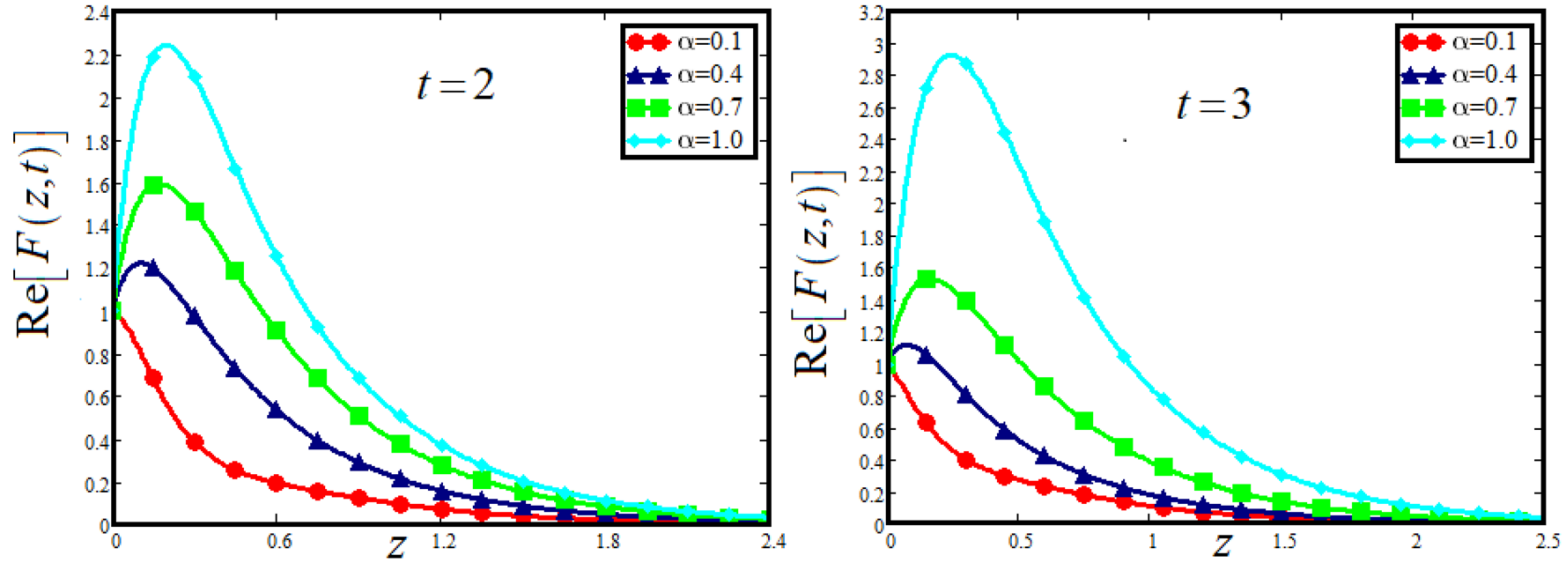
The impact of $\alpha$ and time $t$ on the Jeffery hybrid nanofluid velocity, when $Gr = 80$, $\Pr = 600$, $Nr = 8$, $\phi_{hnf} = 0.03$, $\lambda = 0.5$, $\lambda_{1} = 2.5$ and $r = 1$.

**Figure 7:**
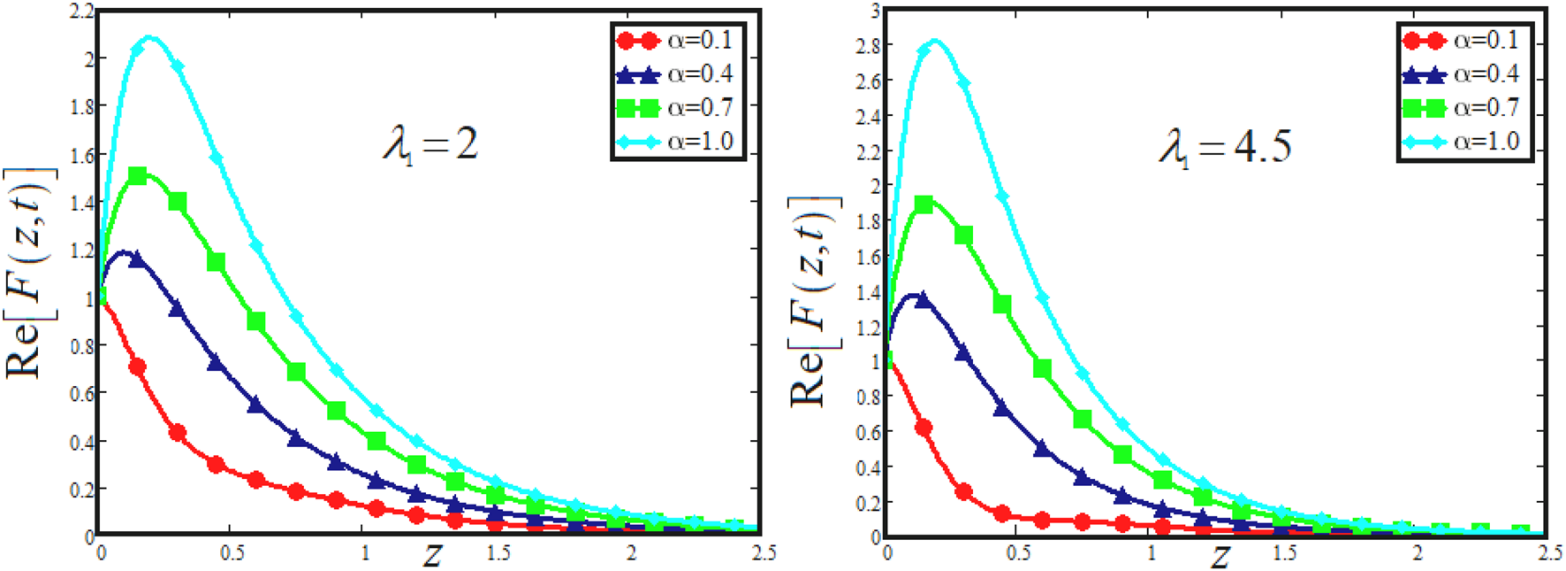
The impact of $\alpha$ and $\lambda_{1}$ on the Jeffery hybrid nanofluid velocity and temperature of Jeffery hybrid nanofluid, when $Gr = 80$, $\Pr = 600$, $Nr = 8$, $t = 2$ , $\lambda = 0.5$, $\phi_{hnf} = 0.03$ and $r = 1$.

**Figure 8:**
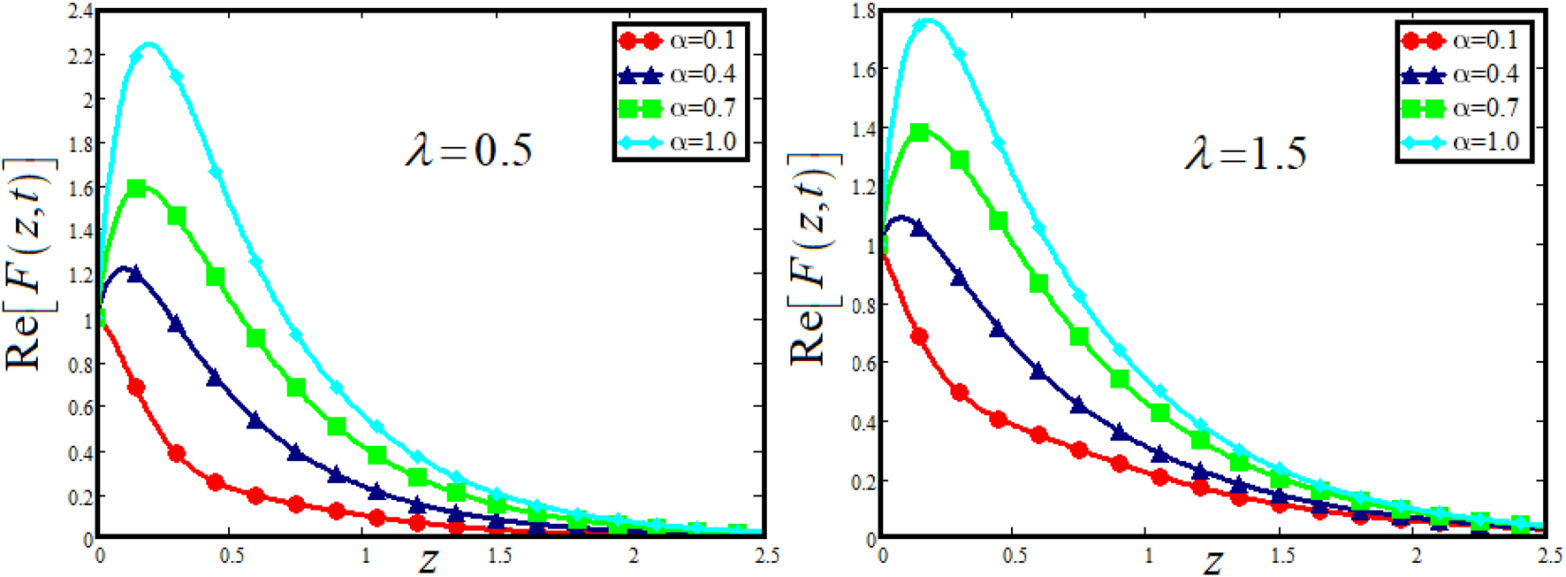
The impact of $\alpha$ and $\lambda$ on the Jeffery hybrid nanofluid velocity and temperature of Jeffery hybrid nanofluid, when $Gr = 80$, $\Pr = 600$, $Nr = 8$, $t = 2$ , $\phi_{hnf} = 0.03$, $\lambda_{1} = 2.5$ and $r = 1$.

**Figure 9:**
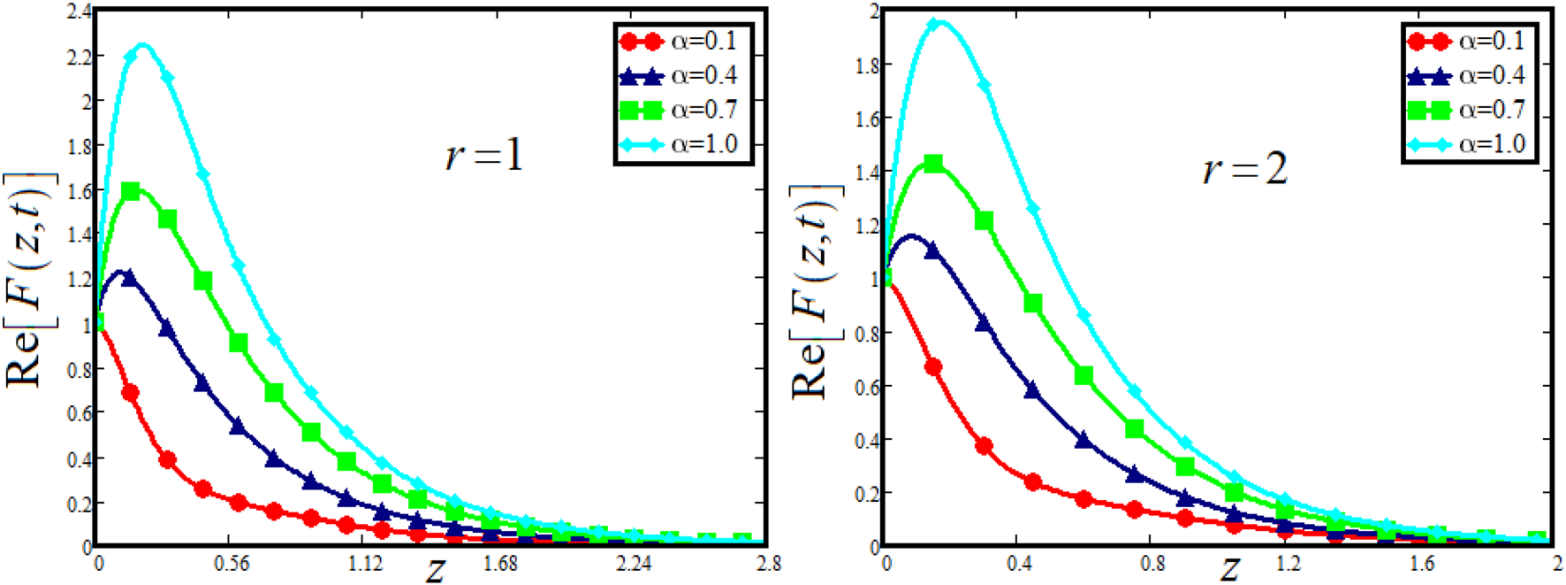
The impact of $\alpha$ and $r$ on the Jeffery hybrid nanofluid velocity and temperature of Jeffery hybrid nanofluid, when $Gr = 80$, $\Pr = 600$, $Nr = 8$, $\phi_{hnf} = 0.03$, $t = 2$, $\lambda = 0.5$ and $\lambda_{1} = 2.5$.

**Figure 10:**
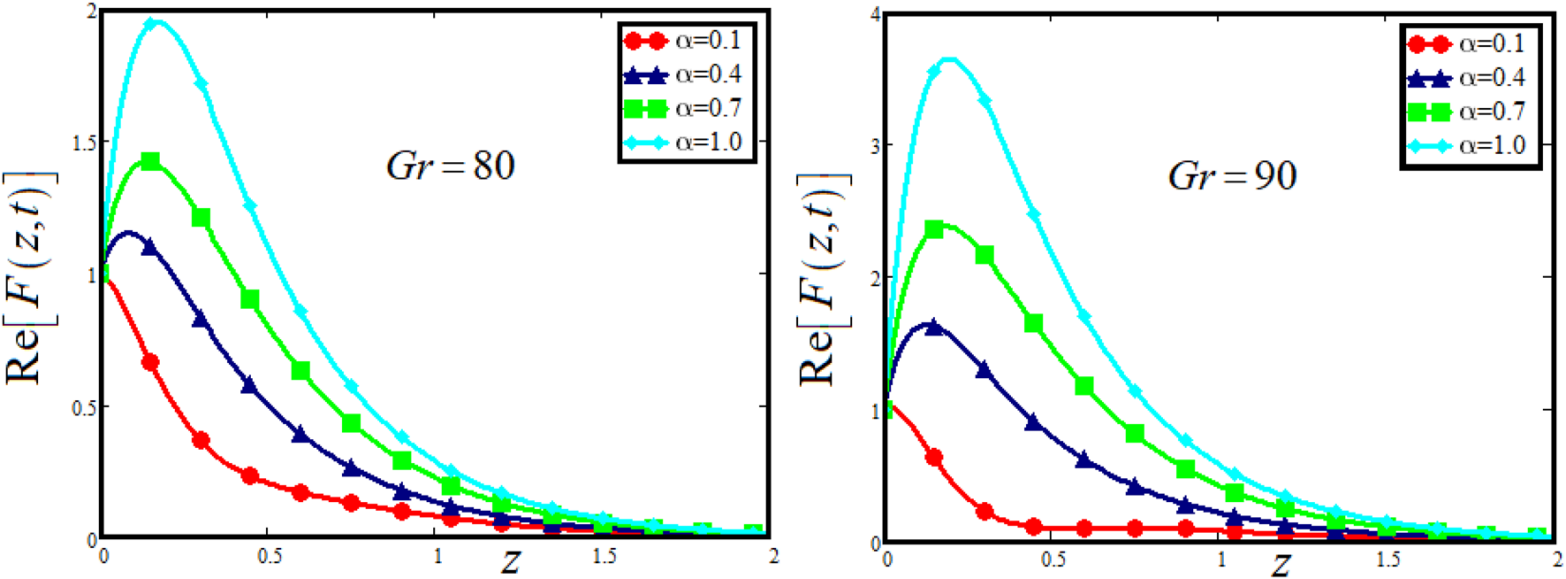
The impact of $\alpha$ and $Gr$ on the Jeffery hybrid nanofluid velocity when $r = 2$, $\Pr = 600$, $Nr = 8$, $\phi_{hnf} = 0.03$, $t = 2$, $\lambda = 0.5$ and $\lambda_{1} = 2.5$.

**Figure 11:**
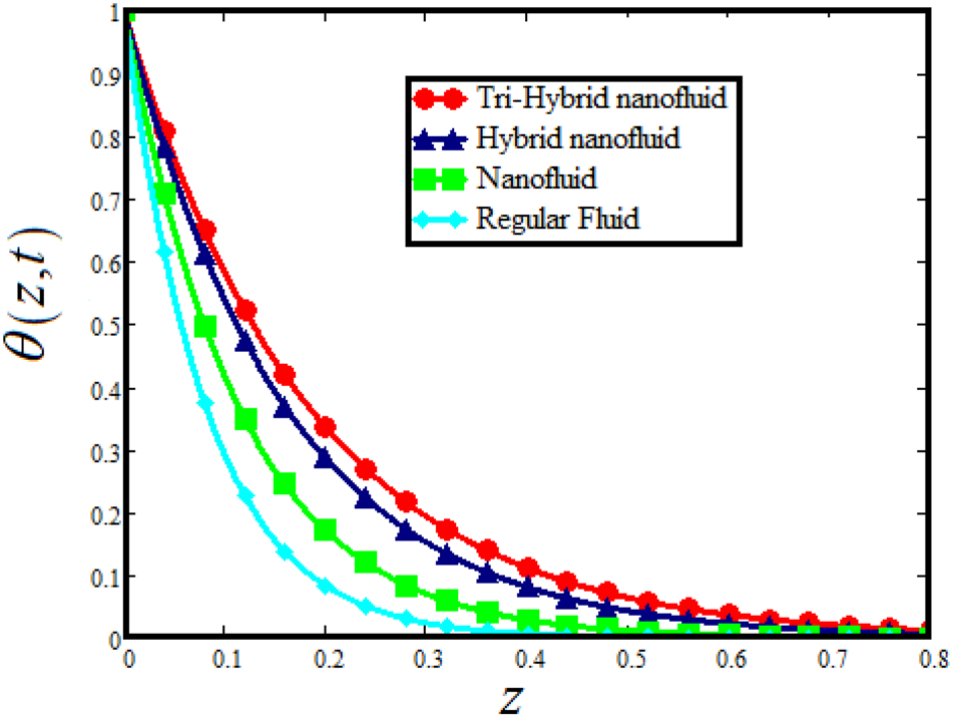
The Comparative analysis of Tri-Hybrid nanofluid with the hybrid nanofluid, mono nanofluid and regular fluid on temperature of the Jeffery fluid.

**Figure 12:**
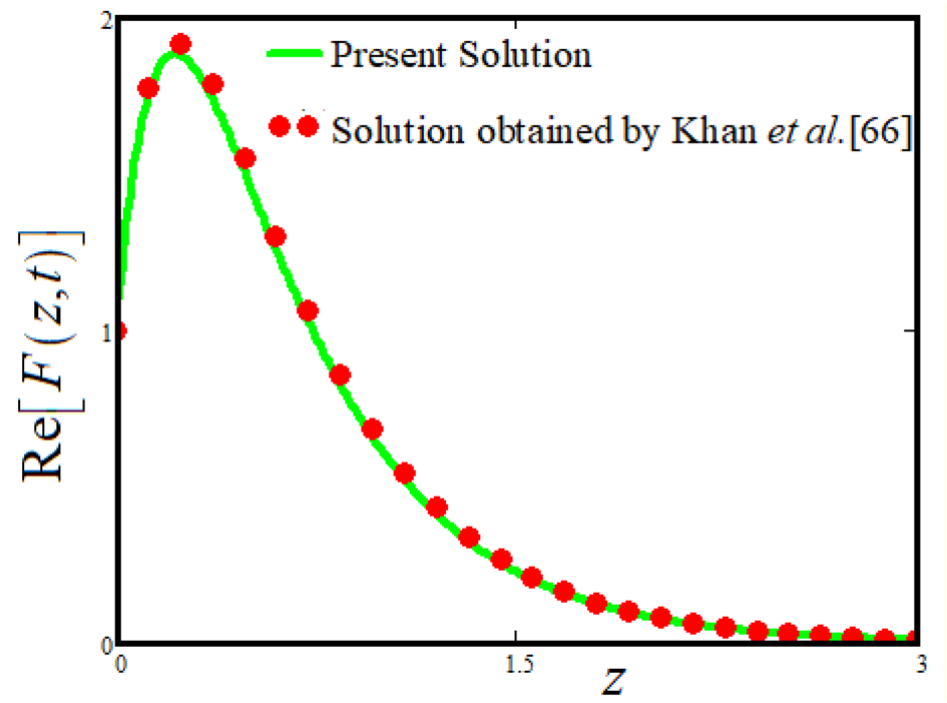
The Comparative analysis of the present solutions with the solutions obtained by Khan et al.^65^ when $r = 0$, $\alpha \to 1$, $\phi_{hnf} = 0$, $Gr = 80$, $\Pr = 600$, $Nr = 8$, $t = 2$, $\lambda = 0.5$ and $\lambda_{1} = 2.5$.

The physical sketch can be visualized in Fig. 1. The flow chart of ternary hybrid nanofluid is highlighted in Fig. 2. This figure presents the preparation method for the mixture of ternary hybrid nanofluid in the engine oil base fluid. The operational framework of the present research work is highlighted in Fig. 3. The influence of $\alpha$ and $\phi_{hnf}$ on the Jeffery tri-hybrid nanofluid velocity and temperature are visualizes in Fig. 4a and b respectively. From Fig. 4a it can be observed that increasing the values of $\alpha$ the Jeffery hybrid nanofluid velocity get higher. This impact on velocity distribution decreases the viscous forces and explain the hidden memory of the fluid flow in a rotating frame. The fluid in a rotating system can be inspected deeply by varying the fractional parameter and it may be used in many physical situations. Furthermore, results of fractional model of Jeffery fluid in a rotating frame can be compared with the classical model by taking $\alpha = 1$. The beauty of fractional model solutions is that it can be compared with experimental values by varying $\alpha$ and can get the appropriate solutions when required. Moreover, at the same time we differ the volume fraction in Fig. 4a for highlighting the impact $\phi_{hnf}$ on the flow profile. This shows an interesting result on the fluid flow in a rotating frame. From the figure on can observed that for higher $\phi_{hnf}$ the flow can be declines in a rotating system. This is because of the concentration of nanoparticles, increasing the $\phi_{hnf}$ concentration get higher as a result there is a resistive force developed which called as viscous forces. These forces make the fluid slow down as a result the velocity get lowered in the boundary layer region. Similarly, from Fig. 4b it can be observed that the higher values of $\alpha$ causes an increment in the heat profile which has many physical uses in modern sciences. The results for heat profile can be changed by varying $\alpha$ according to the temperature required during the heat flow in a rotating system. Moreover, the impact of $\phi_{hnf}$ can be observed on the temperature profile. The temperature of the fluid gets higher for greater values it is due to the fact that higher the nanoparticles concentration the friction forces developed in within the fluid due to which kinetic energy increase which is responsible for the heat increment. The impact of $\alpha$ and $Nr$ on the Jeffery tri-hybrid nanofluid velocity is highlighted in Fig. 5a, where we have shown the impact of $\alpha$ and $Nr$ on the fluid velocity. When radiation get higher the fluid flow in a rotating frame gets accelerated. The $Nr$ increase the velocity, it is since radiation enhances the fluid velocity due to the increase of inner forces which responsible for increase in the fluid velocity. Similarly, the impact of $Nr$ is reported in Fig. 5b, on the temperature profile of the fluid flowing in a rotating frame. In particular, higher values of $Nr$ enhances the flow temperature in a rotating frame, because of increasing the inner energy of the fluid. Analogously, when the energy within the fluid gets higher due to positive values of, temperature increases. The influence of time $t$ is highlighted Fig. 6. From the figure it can be observed that for higher values of $t$, the fluid velocity gets accelerated. The latter happens because we considered the unsteady flow in a rotating frame. Then, for higher values of $t$ the fluid velocity increases and we can note that both the fractional parameter and the time is highlighted in the same time. From the figure we easily recognize $\alpha$ behaves analogously w.r.t. the velocity profile for short and large time. The influence of Jeffery fluid parameter $\lambda_{1}$ is highlighted in Fig. 7 on the velocity profile. The fluid parameter $\lambda_{1}$ shows the ratio of relaxation time and retardation time. In particular, the increase in $\lambda_{1}$ gives us rise in the shear stresses. Therefore, the increase in $\lambda_{1}$ results in an increase in the fluid velocity. The latter is due to the fact that for higher values of $\lambda_{1}$ an immediate response is noted in the applied shear stress which is responsible to accelerate the fluid flow and hence the velocity of the Jeffery fluid increases. Moreover, it is observed from the plot that if we vary $\alpha$ and $\lambda_{1}$ at the same time, we note an increasing of the Jeffery parameter velocity, and the impact of $\alpha$ is the same for small and large value of such a parameter. Similarly, the influence of material parameter $\lambda$ and $\alpha$ is plotted in Fig. 8 on the flow of Jeffry tri-hybrid nanofluid. From the figure it can be observed that the behavior of $\lambda$ is quiet opposite to $\lambda_{1}$. For higher values of $\lambda$ the flow motion decreases. This happens because $\lambda$ shows the time retardation parameter which control the flow motion when we increase $\lambda$. Increasing $\lambda$ results in a delay in the response of shear stresses which make the fluid flow slowdown in the boundary layer region. In this figure we have found the influence of $\alpha$ and $\lambda$ at the same time and one can noticed that the impact of $\alpha$ is same for small and large values of material parameter $\lambda$. Figure 9 visualizes the impact of rotation parameter $r$ and $\alpha$ on the velocity of the Jeffery fluid in a rotating frame. From the figure it can be clearly seen that for higher values of the $r$ velocity get lowered in a rotating frame. When we increase $r$ there is Coriolis forces which built up and have the property to suppress the motion of the flow due to which the velocity retards. The impact of $\alpha$ is same for small and large values of rotation parameter. The impact of $Gr$ on the velocity of Jeffery tri-hybrid nanofluid is highlighted in Fig. 10. From the figure at the same time we can see the impact of $\alpha$ and $Gr$. For the escalating values of $Gr$ the fluid velocity gets higher it is due to the fact that when by increasing $Gr$ the buoyancy forces developed due to which the fluid accelerated in a rotating frame. This is the reason that the fluid velocity increases it is due to the fact that these buoyancy forces are responsible for the accelerating fluid in a rotating frame as a result get higher. The comparative analysis between the mixture of tri-hybrid nanofluid flow in a rotating frame with hybrid nanofluid, nanofluid and classical fluid is highlighted in Fig. 11 for temperature of the fluid. From the comparison it can be noticed that the energy production in the case of tri-hybrid nanofluid is higher as compared to other fluid like hybrid nanofluid, nanofluid and classical fluid. In the present analysis this is the main motivation that tri-hybrid nanofluid performed well as compared to regular fluid, hybrid fluid and nanofluid. The energy production of the system in a rotating frame gets higher for engine oil based tri-hybrid nanofluid. The engine oil efficiency performance is better in the case of tri-hybrid nanofluid. The life span of machinery which uses engine oil is better and performed will and enhance the rate of heat transfer more in the case of regular engine oil. Furthermore, it is worth noting that the rate of heat transfer of the engine oil enhances up-to 18.728% by using the mixture of three different nanoparticles in a single base fluid engine il. This advance class of nanofluid have useful applications in many cooling systems, heat exchangers, heat turbines and other industrial applications. The comparison of the present results with the published work by Khan et al.^65^ is highlighted in Fig. 12. In our obtained solutions we put the rotation parameter $r = 0,$ fractional parameter $\alpha \to 1$ and volume fraction parameter of tri-hybrid nanofluid $\phi_{hnf} = 0$ then our fractional tri-hybrid nanofluid model of Jeffery fluid in a rotating frame reduced to the solution classical solution of Jeffery fluid in the absence of rotation which wes done by Khan et al*.*^65^ which verify our botianed soluitons. This comparison of our solutions to the already published work validate our results.

Thermal properties of nano additives working fluid is listed in the Table 1. The skin friction is highlighted in Table 2 against the variation in different fluid parameters which effect the flow in a rotating frame. From the Table 2 skin friction get higher for greater values of $\alpha$, $Gr$, $Nr$ and $\lambda_{1}$ while the skin friction decreases for the higher values of $\phi_{hnf}$, $\Pr$, $t$, $\lambda$ and $r$. The Nusselt number is highlighted against different parameters in Table 3. From the table Nusselt number increment can be noticed for increasing the values of $\phi_{hnf}$, $t$ and $\alpha$ while the Nusselt number decreases for higher values of radiation parameter $Nr$. The impact of these values on the Nusselt number can be used in many engineering problems. Table 4 shows the Nusselt number variation by varying the volume friction from 0.01 to 0.04. From the table when we increase the volume fraction the Nusselt number increases as result the rate of heat transfer enhances up-to 18.728% in engine oil. This table is very important because it calculated the rate of heat transfer in the working fluids engine oil. When the mixture of three different nanoparticles is added in the working oil, it enhances the rate up-to 18.728%, then naturally increasing the working capability of engine oil.

**Table 1:** Thermo-physical properties of nanoparticles and base fluid^10,54–57^.

Parameters	Engine oil	${\text{CuO}}$	${\text{Al}}_{2} {\text{O}}_{3}$	${\text{TiO}}_{2}$
$\rho \left( {{\text{kg}}/{\text{m}}^{3} } \right)$	884	8933	3970	4250
$k\left( {{\text{W}}/{\text{m}} \cdot {\text{K}}} \right)$	0.144	401	40	8.95928
$C_{p} \left( {{\text{J}}/{\text{kg}} \cdot {\text{K}}} \right)$	1910	385	765	686.2
$\beta \left( {1/{\text{K}}} \right)$	70	1.67	0.00000508	0.9
Shape	–	Spherical	Platelet	Cylindrical

**Table 2:** Skin friction.

$\alpha$	$\phi_{hnf}$	$Gr$	$\Pr$	$Nr$	$t$	$\lambda$	$\lambda_{1}$	$r$	$Sf$
0.5	0.02	80	600	8	1.5	0.5	2.5	1	10.007
**0.8**	0.02	80	600	8	1.5	0.5	2.5	1	14.474
0.5	**0.04**	80	600	8	1.5	0.5	2.5	1	5.869
0.5	0.02	**90**	600	8	1.5	0.5	2.5	1	11.535
0.5	0.02	80	**650**	8	1.5	0.5	2.5	1	9.63
0.5	0.02	80	600	**10**	1.5	0.5	2.5	1	10.973
0.5	0.02	80	600	8	**2**	0.5	2.5	1	9.201
0.5	0.02	80	600	8	1.5	**0.8**	2.5	1	8.474
0.5	0.02	80	600	8	1.5	0.5	**3.5**	1	12.741
0.5	0.02	80	600	8	1.5	0.5	2.5	**2**	8.553

Significant values are in [bold].

**Table 3:** Numerical values for Nusselt number.

$\phi_{hnf}$	$Nr$	$t$	$\alpha$	$Nu$
0.02	7	1.5	0.5	8.01
**0.04**	7	1.5	0.5	8.032
0.02	**10**	1.5	0.5	6.841
0.02	7	**2.2**	0.5	8.543
0.02	7	1.5	**0.8**	8.504

Significant values are in [bold].

**Table 4:** Percentage increase in Nusselt number.

$\phi_{hnf}$	$Nr$	$\Pr$	$\alpha$	$t$	$Nu$	${\text{Percentage}}\,{\text{Enhancement}}$
0.00	7	600	0.5	1.5	55.461	–
0.01	7	600	0.5	1.5	57.981	4.54%
0.02	7	600	0.5	1.5	60.551	9.177%
0.03	7	600	0.5	1.5	63.173	13.9%
0.04	7	600	0.5	1.5	65.848	18.728%

## Conclusion

In this analysis the newly developed fractional operator has been considered. The new operator was derived by combing the constant proportional operator and Caputo fractional derivative to form constant proportional Caputo CPC operator. The CPC operator is applied on the classical model of Jaffery fluid to to transform into fractional model of Jaffry fluid. The tri-hybrid nanofluid was formed by the suspension of three different kinds of nanoparticles in a single bas fluid enginw oil for the advance cooling purposes and had many engineering appplications. In this study the spherical shape ${\text{CuO}}$, platelet shape ${\text{Al}}_{2} {\text{O}}_{3}$ and cylindrical shape ${\text{TiO}}_{2}$ nanoparticles are added in engine oil to form tri-hybrid nanofluid. The impact of new idea of tri-hybrid nanofluid is highlighted on the Jeffry fluid flow over an infinite vertical plate in a rotating frame. All the flow parameterers are highlighted in figures using MATHCAD software. Furthrmote, the engineering values for skin friction and nusselts numbers are calculated for Jeffery fluid in a rotating frame and listed in tablular form.

During the present investigation the authors get some useful and intersting results which are given by:

- The classsical model of Jeffery fluid have been generalized by applying constant proportional Caputo CPC fractional operator which highlight the memory effect in the system.
- The comparative study have been conducted between tri-hybrid nanolfuid, hybrid nanofluid, nanofluid and regular engine oil.
- Three different kinds of nanoaprticles spherical shape ${\text{CuO}}$, platelet shape ${\text{Al}}_{2} {\text{O}}_{3}$ and cylindrical shape ${\text{TiO}}_{2}$ are added in the base fluid engine oil.
- From the comparasion it has been calculated that the energy production in the case of tri-hybrid nanofluid is higher as compared to other fluid like hybrid nanofluid, nanofluid and classical fluid.
- The comparison highlighted that tri-hybrid nanofluid is more effective compared to nanofluid and regular fluid.
- The rate of heat transfer enhaces upto 18.728% by suspending the mixture of three different kinds of nanoparticcles in a singlee base fluid engine oil.
- The thermal performance is higher for tri-hybrid nanofluid in engine oil and improve the effeicecy of the working fluids.
- The Jaffery fluid veocity gets higher for higher values of $\alpha$, $Nr$, $t$ and $\lambda_{1}$.
- The Jaffery fluid velocity declines when increases the values of $\phi_{hnf}$, $\lambda$ and $r$.
- The temperature increases with the increase in $\alpha$, $\phi_{hnf}$ and $Nr$.

## Data Availability

All data used in this manuscript have been presented within the manuscript. No data is hidden or restricted.
